# Scene-Adaptive Line-Aware Visual Measurement Conditioning for Stereo Visual–Inertial Odometry

**DOI:** 10.3390/s26185760

**Published:** 2026-09-10

**Authors:** Yi Liang, Bingbing Hang, Wenqiang Li, Yue Yuan, Feng Shen

**Affiliations:** 1 School of Instrumentation Science and Engineering, Harbin Institute of Technology, Harbin 150001, China; 22b901037@stu.hit.edu.cn (Y.L.); wen.q.li@foxmail.com (W.L.); 2 Department of Industrial and Systems Engineering, The Hong Kong Polytechnic University, Hong Kong, China; yuena.yuan@polyu.edu.hk

**Keywords:** visual–inertial odometry, visual measurement conditioning, Multi-State Constraint Kalman Filter, structural priors, stereo vision

## Abstract

Accurate stereo visual–inertial measurement is essential for mobile robots operating in Global Navigation Satellite System (GNSS)-denied and structurally complex environments. In stereo visual–inertial odometry (VIO), pose and trajectory outputs depend strongly on the point measurements delivered by the visual front end before sensor-fusion update. In sparse-texture but structurally regular scenes, tracked point features may exhibit poor persistence, uneven spatial distribution, and local tracking noise, even when informative line structures are present. Existing point–line VIO methods can improve positioning accuracy by introducing line landmarks or line residuals, but they usually modify the estimator state, measurement model, and Jacobian treatment. We present a scene-adaptive line-aware visual measurement conditioning method for stereo VIO front ends with point-measurement updates. The method uses 2-D image-line segments as lightweight structural priors and applies bounded normal-direction conditioning to reliable point measurements before a fixed-interface VIO back-end update. A sparse pruning safeguard removes only highly inconsistent long-lived tracks under strong structural support, while a scene-level confidence gate attenuates the intervention when line evidence is weak or unstable. The method is instantiated and evaluated in an S-MSCKF pipeline. On the reported EuRoC MAV sequences, it reduces the sequence-averaged absolute trajectory error (ATE) RMSE by approximately 13% relative to S-MSCKF, with 3–27% reductions on machine-hall sequences. On three real-world robot measurement sequences with an RTK-aided inertial reference, the mean Sim(2)-aligned planar position error decreases from 8.72 m to 7.31 m, and the mean yaw error decreases from 8.02° to 6.76°; an additional scale-preserving SE(2) evaluation reveals sequence-dependent planar behavior and residual metric-scale sensitivity. Candidate-level stereo-consistency diagnostics show subpixel mean and 95th-percentile image-domain perturbations without systematic vertical-stereo bias, while the final reliability-weighted primary-view update is analytically bounded by approximately 0.221 pixels in the reported implementation. Runtime profiling reports an average front-end time of 33.34 ms on the tested CPU platform, close to the 33.3 ms frame period of the 30 Hz stereo input, although the μ+3σ runtime of 47.23 ms exceeds a strict frame-by-frame 30 Hz budget. These results suggest that line-aware front-end conditioning can improve visual measurement quality in structured stereo visual–inertial sensing without modifying the evaluated back-end interface.

## 1. Introduction

Reliable stereo visual–inertial measurement is fundamental for mobile robots, unmanned aerial vehicles, and other autonomous systems operating in Global Navigation Satellite System (GNSS)-denied environments, including cooperative, aerial, and marine robotic applications that depend on onboard perception and navigation [1,2,3]. By tightly coupling camera and inertial measurements, visual–inertial odometry (VIO) provides high-rate estimates of platform pose and trajectory when external positioning infrastructure is unavailable [4,5,6,7,8]. In this article, the measurement output of interest is the VIO-derived pose trajectory, and the front-end image observations are treated as visual measurements that determine the quality of the subsequent sensor-fusion update. For stereo VIO systems with point-measurement front ends, measurement reliability depends strongly on the quality, persistence, and spatial distribution of tracked visual observations, especially in scenes with sparse texture, locally clustered features, or uneven feature coverage [9,10,11]. The Multi-State Constraint Kalman Filter (MSCKF) is a classical filtering framework for visual–inertial estimation and remains a representative choice for stereo VIO systems that require fixed state size, predictable computational cost, and a clear point-measurement update interface [9,10,12,13]. For this reason, S-MSCKF is used as the evaluation back end in this study.

In a typical stereo VIO front end, point features are detected and tracked using corner detectors and optical flow [9,10,14,15]. Stereo matching, RANSAC-based outlier rejection, lifespan-based pruning, and grid-based feature management are then used to maintain point observations for the back-end update [9,10]. This design is effective when sufficient texture and parallax are available. In structured corridors, industrial environments, and weakly textured scenes, however, point features may become locally clustered, short-lived, or affected by residual tracking errors. Under these conditions, the visual measurements delivered to the back end can be weakened even if the state-estimation formulation remains unchanged. The resulting measurement problem is to improve the quality and geometric consistency of point observations before they enter the sensor-fusion update.

Line structures provide complementary geometric information in man-made environments. Compared with isolated point features, straight-line segments often exhibit stronger spatial regularity and better cross-frame consistency [16,17,18,19]. Existing point–line simultaneous localization and mapping (SLAM) and VIO systems exploit this property by explicitly modeling 3-D lines and introducing point–line or line–line reprojection residuals into the back end [17,18,19,20,21]. These methods can enhance geometric constraints in line-rich scenes, but they usually require explicit line parameterization, additional residual models, and nontrivial Jacobian or observability treatment. The added modeling requirements make direct integration into lightweight MSCKF-style filtering pipelines less straightforward.

For stereo-inertial sensing systems, a complementary route is to use image-line cues at the front end to regularize point observations before fusion. This route can exploit structural information while keeping the estimator formulation unchanged, but line guidance should not be activated indiscriminately. Detected line segments may be unreliable under low parallax, rapid motion, transient edges, or weakly persistent structures. Over-activation of unreliable line guidance may disturb feature-track persistence and weaken multi-view constraints. Effective line-aware front-end processing should therefore be scene-adaptive, bounded, and selective. Despite the potential of this route, exploiting 2-D line cues to improve point-measurement quality before a fixed-interface VIO update has received limited attention.

To address this gap, we develop a scene-adaptive line-aware visual measurement conditioning module for stereo VIO front ends. The module uses 2-D image-line segments as structural priors for image-domain point-measurement regularization, thereby improving local geometric consistency while maintaining a standard point-measurement interface to the back end. In this article, the module is instantiated in an S-MSCKF pipeline and evaluated through pose-trajectory errors against public ground truth and an RTK-aided inertial reference, with additional diagnostics used to characterize candidate-level stereo geometry and the final boundedness of the conditioning layer.

The main contributions of this article can be summarized as follows.

1. We present a line-aware visual measurement conditioning design for stereo VIO front ends that use point-measurement updates. Image-plane line-segment priors are used only at the feature-management stage, with no change to the state representation, propagation model, or stereo point-measurement interface of the evaluated fixed-interface back end.
2. We design a reliability-driven activation mechanism that combines segment-level structural quality with temporally smoothed scene-level confidence, so that line guidance is emphasized in reliable structured scenes and attenuated under unstable or weak line support.
3. We formulate a bounded intervention rule that applies normal-direction conditioning to reliable point observations and uses pruning only as a safeguard for highly inconsistent long-lived tracks under strong structural evidence, while preserving the original stereo measurement interface.
4. We instantiate the proposed front end in an S-MSCKF pipeline and evaluate it on the EuRoC MAV dataset and real-world robot measurement sequences with an RTK-aided inertial reference, reporting trajectory-error metrics, runtime performance, candidate-level stereo-consistency diagnostics, and diagnostic statistics that explain both accuracy gains and degradation cases.

The remainder of this article is organized as follows. Section 2 reviews related work on VIO, line-feature-based SLAM/VIO, and front-end feature-quality assessment. Section 3 presents the system overview. Section 4 describes the proposed scene-adaptive line-aware front-end module. Section 5 reports the experimental results and diagnostic analysis. Section 6 concludes this article.

## 2. Related Work

### 2.1. Visual–Inertial Odometry Back Ends

Visual–inertial odometry (VIO) has mainly developed along two back-end paradigms: recursive filtering and nonlinear optimization [4,6,8]. Filter-based methods fuse visual and inertial information recursively with compact state representations and predictable computational complexity, while recent navigation filters further explore invariant-error formulations and adaptive covariance modeling for robust multi-source fusion [22]. The Multi-State Constraint Kalman Filter (MSCKF) is a representative filtering framework that removes feature states from the filter update and exploits multi-state constraints for efficient visual–inertial estimation [12]. Stereo extensions and practical implementations have further improved the robustness and deployment of MSCKF-style pipelines [9,10]. ROVIO and VINS-Mono represent filter-based and sliding-window optimization-based VIO routes, respectively, and provide useful references for comparing estimator designs [5,6].

Compared with optimization-based systems, MSCKF-style filters are less flexible in residual construction but remain attractive when a fixed state size, stable latency, and simpler deployment are required. For such fixed-state filters, front-end measurement quality is a primary lever for robustness improvement. Across VIO back ends, estimation accuracy depends strongly on feature detection, tracking, matching, and outlier rejection [7,11]. Classical MSCKF front ends typically rely on corner-like point features, optical-flow tracking, stereo matching, grid-based management, and lifespan-based pruning [10,14,15]. Learned features and matchers can improve repeatability and matching robustness [23,24,25], but their integration into real-time MSCKF pipelines remains nontrivial. Front-end feature quality therefore remains central to robust fixed-state visual–inertial filtering.

### 2.2. Line Features in SLAM and VIO

Line structures are common in man-made environments and provide geometric information complementary to isolated point features. Straight-line segments often exhibit stronger spatial regularity and cross-frame consistency in corridors, indoor scenes, and structured outdoor environments [16,17,18,19]. Efficient 2-D line detectors and descriptors have made image-plane line segments accessible to visual pipelines [26,27,28]. Recent measurement-oriented VIO studies in *Measurement Science and Technology* have investigated structural-line-aided wheeled-robot localization and adaptive line extraction for faster point–line VIO under low-texture or illumination-changing conditions [19,21]. More recent point–line VIO systems further improve line matching, line-feature processing, and hybrid point–line estimation efficiency [29,30,31]. These studies support the value of line features for visual–inertial sensing, but they mainly use line observations within point–line VIO formulations.

Existing point–line SLAM/VIO methods mainly exploit these structural primitives through explicit back-end modeling [17,18,19,20,29,30,31]. They can improve geometric constraints in line-rich scenes, especially when stable structural elements are repeatedly observed. However, line information is usually coupled with estimator-specific parameterization, residual construction, covariance modeling, and update procedures. This coupling is natural in optimization or mapping-oriented formulations, but it is less direct for fixed-state MSCKF pipelines.

Line observations have also been used to guide feature detection, data association, or mapping while keeping the system largely point-based [16,18]. Nevertheless, many such approaches still absorb structural information into map representation, graph constraints, or higher-level data association. This leaves a narrower gap: using line features as bounded image-domain conditioning cues for point observations before a fixed-interface VIO update. In the proposed module, a point is not retained merely because it lies near a line, nor is it forced to satisfy a hard point–line constraint. Line support is used only when segment quality, point support, directional consistency, and scene-level confidence jointly indicate reliable structural evidence, and the resulting displacement is explicitly bounded.

Table 1 summarizes the main technical differences among representative point-only front ends, explicit point–line VIO/SLAM, measurement-oriented line-feature VIO studies, and point-based front-end adaptation. The comparison shows that most line-aware VIO/SLAM methods use line structures as estimator or mapping constraints, whereas the proposed method uses line support only as bounded conditioning of point measurements before an unchanged point-measurement VIO update. The contribution is a reliability-gated measurement-conditioning mechanism rather than a new point–line estimator: it converts 2-D structural support into a small, interface-preserving perturbation of existing point observations.

### 2.3. Front-End Adaptation and Feature Quality Assessment

Front-end robustness has been widely studied through feature-quality assessment and adaptive feature selection. Existing methods commonly evaluate point features according to gradient strength, corner response, tracking lifespan, spatial distribution, or matching consistency, and then retain features expected to provide stable constraints [7,10]. Semantic and geometric cues, such as dynamic-object masks, planar regions, or static-region priors, have also been used to suppress features from unreliable regions and emphasize stable scene components [32]. These strategies improve robustness by suppressing or correcting unreliable point observations, but they usually remain centered on point-wise criteria.

Most front-end adaptation methods judge each feature according to its own appearance, motion history, or semantic region, whereas the persistent relationship between feature observations and structural elements is less explicitly exploited. This relation is important for fixed-interface VIO back ends that rely on multi-frame point tracks: unstable, short-lived, or poorly distributed tracks can weaken the effective multi-view constraints even if the back end is unchanged. Using structural information for feature regulation therefore requires a mechanism that improves local measurement consistency while preserving the standard stereo point-measurement interface.

Prior work leaves a gap between point-centered VIO front ends and line-aware systems with more involved back-end formulations. Existing point–line VIO methods typically introduce line information as additional estimation variables or residual constraints. In contrast, the proposed method does not estimate line landmarks or construct line residuals. Detected 2-D line segments are used only as front-end structural priors to condition existing point observations before they enter the original stereo point-measurement interface. The contribution therefore lies in reliability-gated measurement conditioning rather than in a new point–line estimator.

## 3. System Overview

The system is formulated as a pre-update visual measurement-conditioning layer for stereo VIO pipelines that consume tracked stereo point measurements. After the standard stereo visual tracking pipeline provides candidate point tracks, the line-aware layer is inserted before the final feature-retention and measurement-packaging stage. In the implementation evaluated in this paper, the conditioned measurements are passed to an S-MSCKF back end, whose inertial propagation, camera-state augmentation, feature elimination, and update follow the baseline formulation.

**Figure 1:**
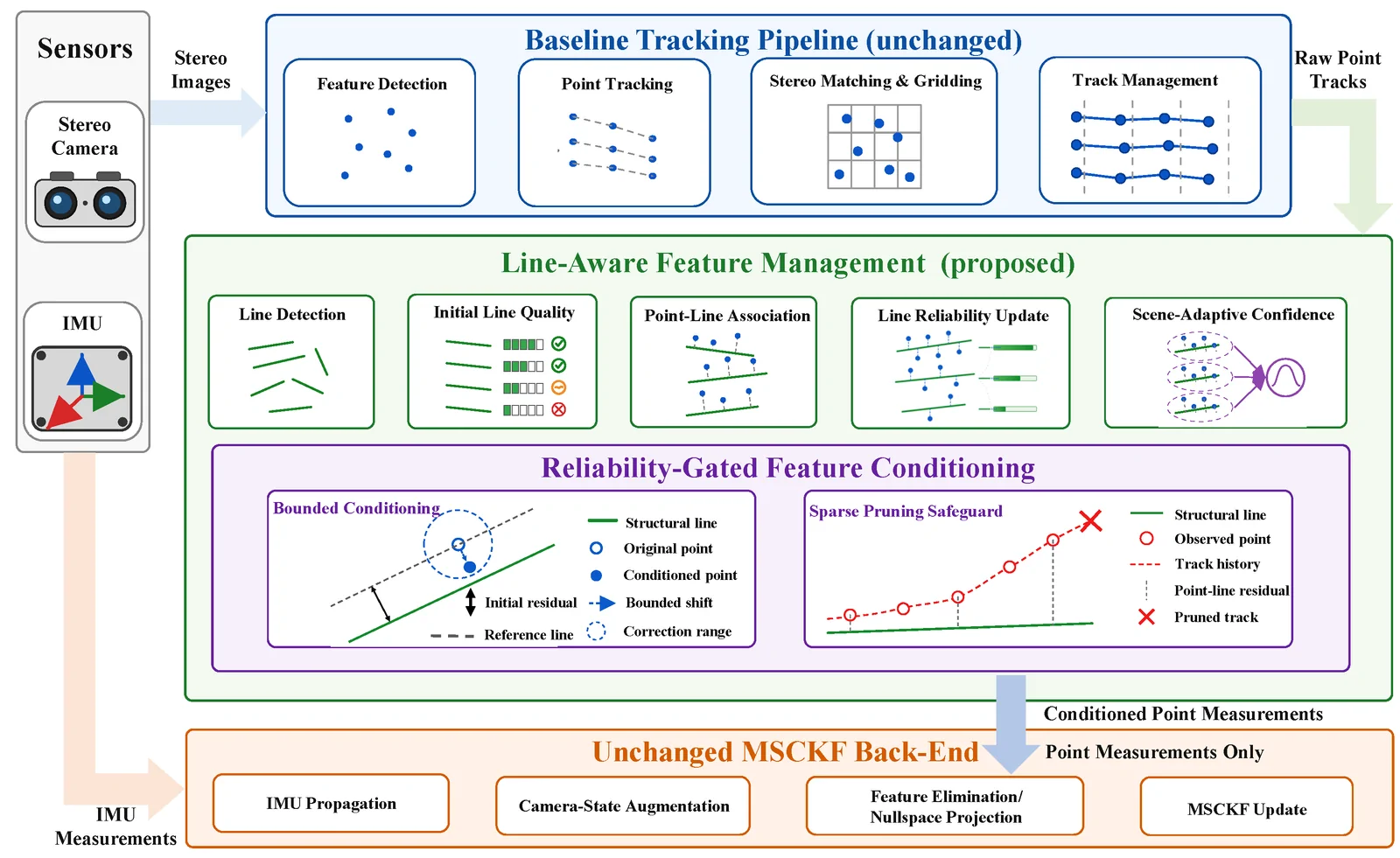
Overview of the proposed line-aware visual measurement-conditioning pipeline.

As illustrated in Figure 1, 2-D line segments are extracted from the primary image, i.e., the left image in the stereo pair, and treated as image-domain structural primitives. Their image-level evidence, geometric point–line association, point-observation support, and directional consistency jointly form a line-supported reliability estimation pipeline. The estimated reliability is then summarized by a scene-level confidence, which regulates a unified front-end intervention consisting of bounded primary-view conditioning and sparse pruning. In line-supported scenes, the layer improves the local consistency of conditioned point observations; under weak or unstable line evidence, the front end smoothly reverts toward the original point-based behavior.

The output remains a standard stereo point-measurement set for the downstream VIO back end. Structural guidance is decoupled from stereo correspondence estimation: line cues regulate the primary-view measurement coordinate and retention decision, while the stereo measurement format is preserved. The raw-to-conditioned measurement mapping is introduced in Section 4.1. This mapping converts scene-dependent line support into bounded point-measurement conditioning before filtering and improves the visual measurements supplied to the S-MSCKF implementation used for evaluation without changing its sensor-fusion interface.

## 4. Scene-Adaptive Line-Aware Visual Measurement Conditioning

The module is an interface-preserving front-end measurement layer placed before a stereo VIO back-end update. It consists of three coupled stages: line-supported reliability estimation, scene-level intervention regulation, and bounded point conditioning with sparse pruning as a safeguard. In the S-MSCKF instantiation used for evaluation, the state representation, propagation model, and stereo point-measurement interface remain unchanged. All scalar design constants are fixed once for the complete evaluation and are not adjusted for individual sequences. The method is described in terms of gate ordering, boundedness, and fallback behavior.

### 4.1. Stereo Measurement Interface and Front-End Regularization

We formulate the module as a pre-update conditioning layer for tracked stereo point measurements. The inertial propagation, camera-state augmentation, feature elimination, state dimension, projection model, and MSCKF update follow the baseline formulation in the evaluated S-MSCKF implementation. The module operates on tracked point observations in the image domain before the final stereo measurement set is formed for the back-end update.

In simple terms, the baseline front end first produces a matched left–right point observation. The proposed layer leaves the right-camera match unchanged and, only when reliable structural-line evidence is available, applies a small bounded correction to the left-image coordinate. Unassociated or unreliable points remain unchanged, while only strongly inconsistent long-lived tracks may be removed. The resulting observation therefore retains the same stereo point-measurement format as the baseline.

The principal notation used in the formulation is summarized in Table 2.

For the *k*-th frame, let $I_{k}^{\mathrm{raw}}$ denote the set of feature IDs after baseline point tracking, stereo matching, and standard outlier checking. For each feature $i\in I_{k}^{\mathrm{raw}}$, the raw stereo point observation is

(1) $$z_{k}^{i,\mathrm{raw}}=\left[\begin{array}{c} p_{0,k}^{i,\mathrm{raw}}\\ p_{1,k}^{i,\mathrm{raw}} \end{array}\right]={\left[\begin{array}{cccc} u_{0,k}^{i,\mathrm{raw}} & v_{0,k}^{i,\mathrm{raw}} & u_{1,k}^{i,\mathrm{raw}} & v_{1,k}^{i,\mathrm{raw}} \end{array}\right]}^{⊤},$$

where $p_{0,k}^{i,\mathrm{raw}}$ and $p_{1,k}^{i,\mathrm{raw}}$ are the undistorted image coordinates of the same matched feature in the left and right cameras, respectively. The superscript “raw” denotes the baseline front-end output before line-aware conditioning.

The line-aware module outputs a retained feature set ${\tilde{I}}_{k}\subseteq I_{k}^{\mathrm{raw}}$ and a conditioned stereo observation:

(2) $${\tilde{z}}_{k}^{i}=\left[\begin{array}{c} {\tilde{p}}_{0,k}^{i}\\ p_{1,k}^{i,\mathrm{raw}} \end{array}\right],\;{\tilde{p}}_{0,k}^{i}=p_{0,k}^{i,\mathrm{raw}}+\delta_{i,k},\;i\in {\tilde{I}}_{k}.$$

Here, $\delta_{i,k}\in R^{2}$ is the bounded primary-view displacement. If line guidance is inactive or unreliable, $\delta_{i,k}=0$. If a feature is rejected by conservative pruning, its index is removed from ${\tilde{I}}_{k}$ and handled as a baseline front-end outlier. The final measurement set for the back-end update is

(3) $${\tilde{Z}}_{k}={\left\{\left(i,{\tilde{z}}_{k}^{i}\right)\right\}}_{i\in {\tilde{I}}_{k}}.$$

This set preserves the stereo point-measurement dimension and feature-ID structure of the baseline interface. Since line segments are extracted only from the primary image, the right-camera observation $p_{1,k}^{i,\mathrm{raw}}$ remains the pre-conditioning stereo match. This choice keeps the accepted stereo correspondence produced by the baseline matcher and avoids constructing a second, line-shifted right-view observation from primary-view line evidence. The conditioned point is therefore a bounded front-end regularization, not an additional probabilistic line constraint or re-triangulated stereo correspondence. Equivalently,

(4) $${\tilde{z}}_{k}^{i}=z_{k}^{i,\mathrm{raw}}+\left[\begin{array}{c} \delta_{i,k}\\ 0_{2} \end{array}\right],\;{∥\delta_{i,k}∥}_{2}\leq \Delta_{0},$$

where the right-camera component is unchanged. No line-measurement likelihood, line residual, line-noise model, or additional Kalman update term is introduced. The conditioned observation retains the baseline S-MSCKF point-measurement covariance: the front-end layer changes the measurement coordinate but does not reduce the assigned visual measurement covariance or otherwise increase filter confidence through covariance scaling. Explicit propagation of line-detection and point–line association uncertainty into a conditioning-aware covariance model is left for future work. The final primary-view update is bounded by (4), and candidate-level stereo-consistency diagnostics are reported in Section 5.2.5.

Because only the primary-view coordinate is modified, the operation can be interpreted as adding a small bounded perturbation to the stereo disparity. For a rectified stereo pair with horizontal disparity $d_{k}^{i}=u_{0,k}^{i}-u_{1,k}^{i}$, the conditioned disparity becomes ${\tilde{d}}_{k}^{i}=d_{k}^{i,\mathrm{raw}}+\delta_{u,i,k}$, where $\delta_{u,i,k}$ is the horizontal component of $\delta_{i,k}$. Under the standard stereo depth relation $Z=f_{x}b/d$, the first-order relative depth perturbation satisfies

(5) $$\frac{\left|\Delta Z\right|}{Z}\approx \frac{|\delta_{u,i,k}|}{|d_{k}^{i,\mathrm{raw}}|}.$$

This relation means that far points with small disparity are more sensitive to primary-view conditioning. Here, $\delta_{u,i,k}$ denotes the horizontal component of the actual final update $\delta_{i,k}$ in (2); it is distinct from the candidate-level diagnostic quantity $\Delta u_{\mathrm{cand}}$ reported in Section 5.2.5. The proposed front end does not assume that the stereo geometry is unchanged; instead, it treats the final primary-view update as a small disparity perturbation and limits this perturbation by design. Candidate-level stereo geometry is inspected separately in the diagnostic analysis. This interpretation also explains why weak-parallax or distant line responses are treated as boundary cases in the diagnostic analysis.

The primary-view displacement is applied in an open-loop image-domain manner. The conditioned coordinate ${\tilde{p}}_{0,k}^{i}$ is used only for current-frame measurement packaging, whereas the optical-flow tracker continues from its raw state. Point–line residuals and line-consistency statistics are computed before conditioning to avoid circular dependence.

The remaining subsections describe the three coupled stages of the front-end layer: line-supported reliability estimation, scene-level intervention regulation, and unified bounded conditioning with sparse safeguard. The implementation sequence is summarized in Table 3.

### 4.2. Line-Supported Reliability Estimation

This subsection estimates whether a current image-line segment provides reliable structural support for nearby point observations. Image evidence initializes line quality, geometric association links raw primary-view observations to nearby segments, track persistence updates reliability, and directional consistency softly regulates line reliability when a stable orientation prior is available. The output is the final line reliability $q_{\mathrm{final},j,k}$.

#### 4.2.1. Image-Evidence Line Quality

Line extraction and reliability initialization are performed in the primary image plane. In frame *k*, each detected line segment $L_{j,k}$ is represented by

$$L_{j,k}≜\left\{a_{j,k},b_{j,k},\ell_{j,k},{\bar{g}}_{j,k},\theta_{j,k},q_{\mathrm{img},j,k}\right\},$$

where $a_{j,k}=\left(x_{j,1,k},y_{j,1,k}\right)$ and $b_{j,k}=\left(x_{j,2,k},y_{j,2,k}\right)$ are endpoints, $\ell_{j,k}$ is length, ${\bar{g}}_{j,k}$ is mean along-line gradient magnitude, $\theta_{j,k}\in \left[0,\pi \right)$ is the folded orientation, and $q_{\mathrm{img},j,k}\in \left[0,1\right]$ is the image-evidence quality.

Given a grayscale image $I_{k}$, horizontal and vertical gradients $G_{x,k}$ and $G_{y,k}$ are computed using the Sobel operator, and the gradient-magnitude map is obtained as

(6) $$G_{k}=\sqrt{G_{x,k}^{2}+G_{y,k}^{2}}.$$

Candidate line segments are detected on the primary image $I_{k}$ using the OpenCV Fast Line Detector (FLD). The detector is used only to provide candidate 2-D segments and is not a contribution of this work. The proposed contribution begins after segment extraction and lies in line-quality assessment, point-supported reliability estimation, scene-adaptive confidence, and bounded point-measurement conditioning. The FLD configuration used in all reported experiments is summarized later with the fixed implementation settings.

For each detected segment $L_{j,k}$, the pixel length is computed as

(7) $$\ell_{j,k}=\sqrt{{\left(x_{j,2,k}-x_{j,1,k}\right)}^{2}+{\left(y_{j,2,k}-y_{j,1,k}\right)}^{2}}.$$

Segments satisfying $\ell_{j,k}<\ell_{\min}$ are discarded. To characterize local edge strength, points ${\left\{q_{j,m,k}\right\}}_{m=1}^{M_{j,k}}$ are uniformly sampled along $L_{j,k}$ with an approximate fixed sampling interval $s_{g}$. The number of samples is defined as

(8) $$M_{j,k}=\max\;\left(5,\left\lfloor \frac{\ell_{j,k}}{s_{g}}\right\rfloor \right).$$

The mean along-line gradient magnitude is then computed as

(9) $${\bar{g}}_{j,k}=\frac{1}{M_{j,k}}\sum_{m=1}^{M_{j,k}}G_{k}\left(q_{j,m,k}\right),$$

where $G_{k}\left(q_{j,m,k}\right)$ is obtained by bilinear interpolation when $q_{j,m,k}$ falls at a noninteger pixel location. Let $\ell_{\mathrm{low},k}$ and $g_{\mathrm{low},k}$ denote the minimum retained length and the minimum retained mean gradient in frame *k*, and let $\ell_{90,k}$ and $g_{90,k}$ denote the corresponding 90th-percentile values. The normalized scores are defined as

(10) $$\begin{array}{cc} {\tilde{\ell }}_{j,k} & =\mathrm{clip}\;\left(\frac{\ell_{j,k}-\ell_{\mathrm{low},k}}{\max(\ell_{90,k}-\ell_{\mathrm{low},k},\varepsilon_{\mathrm{norm}})},0,1\right),\\ {\tilde{g}}_{j,k} & =\mathrm{clip}\;\left(\frac{{\bar{g}}_{j,k}-g_{\mathrm{low},k}}{\max(g_{90,k}-g_{\mathrm{low},k},\varepsilon_{\mathrm{norm}})},0,1\right). \end{array}$$

The image-evidence quality of $L_{j,k}$ is then defined as

(11) $$q_{\mathrm{img},j,k}=\frac{1}{2}{\tilde{\ell }}_{j,k}+\frac{1}{2}{\tilde{g}}_{j,k},\;q_{\mathrm{img},j,k}\in \left[0,1\right].$$

Finally, the segment orientation is computed and folded into [0,π) as

(12) $$\theta_{j,k}=\mathrm{mod}\;\left(atan2(y_{j,2,k}-y_{j,1,k},\;x_{j,2,k}-x_{j,1,k}),\pi \right).$$

Here, mod(·,π) denotes the representative angle folded into [0,π). The tuple $L_{j,k}$ is then passed to association and support-driven reliability updating.

#### 4.2.2. Geometric Point–Line Association and Track-Level Support

Point–line association is evaluated before conditioning. Raw primary-view tracks are associated with current-frame line segments to form support sets, which are then used to obtain $q_{\mathrm{final},j,k}$ and update scene-level confidence.

Let $I_{k}^{\mathrm{raw}}$ be the set of tracked stereo feature IDs before line-aware conditioning, and let $p_{0,k}^{i,\mathrm{raw}}$ denote the raw primary-view coordinate of feature *i*. The detected line-segment set after initial length filtering and image-evidence scoring is denoted by

(13) $$L_{k}={\left\{L_{j,k}\right\}}_{j\in J_{k}},$$

where $J_{k}$ is the corresponding line-index set. For a line segment $L_{j,k}$ with endpoints $a_{j,k}$ and $b_{j,k}$, define

(14) $$v_{j,k}=b_{j,k}-a_{j,k}.$$

The projection parameter of point $p_{0,k}^{i,\mathrm{raw}}$ onto the supporting line of $L_{j,k}$ is

(15) $$t_{i,j,k}=\frac{{\left(p_{0,k}^{i,\mathrm{raw}}-a_{j,k}\right)}^{⊤}v_{j,k}}{∥v_{j,k}{∥}_{2}^{2}}.$$

For projections within the finite segment, the projected point and point–line residual are

(16) $$\pi_{i,j,k}=a_{j,k}+t_{i,j,k}v_{j,k},\;d_{i,j,k}={∥p_{0,k}^{i,\mathrm{raw}}-\pi_{i,j,k}∥}_{2}.$$

The candidate line-index set for feature *i* is

(17) $$\begin{array}{cc} C_{i,k}=\{j\in J_{k}\;|\; & 0\leq t_{i,j,k}\leq 1,\\ & d_{i,j,k}\leq d_{\mathrm{assoc}},\\ & q_{\mathrm{img},j,k}\geq q_{\mathrm{assoc}}\}. \end{array}$$

This gate prevents weak image responses from being promoted solely by geometric proximity.

The set of associated feature IDs is

(18) $$I_{k}^{\mathrm{assoc}}=\left\{i\in I_{k}^{\mathrm{raw}}|C_{i,k}\neq \emptyset \right\}.$$

For $i\in I_{k}^{\mathrm{assoc}}$, the associated segment is selected by the nearest-residual rule:

(19) $$j^{★}\left(i,k\right)=\arg\min_{j\in C_{i,k}}d_{i,j,k},\;d_{i,k}=d_{i,j^{★}\left(i,k\right),k}.$$

For $i\notin I_{k}^{\mathrm{assoc}}$, $j^{★}\left(i,k\right)$ is not defined and the feature is treated as unassociated. The support set of segment $L_{j,k}$ is

(20) $$A_{j,k}=\left\{i\in I_{k}^{\mathrm{assoc}}|j^{★}\left(i,k\right)=j\right\}.$$

The association uses raw primary-view coordinates to avoid circular dependence with conditioning. In the evaluated implementation, subsequent scene-confidence estimation uses the current point–line distance, current line reliability, and feature-track lifetime; no additional point–line persistence counter is used as an active gate.

#### 4.2.3. Support-Driven Reliability from Persistent Point Tracks

The image-evidence score $q_{\mathrm{img},j,k}$ may favor transient edges with strong gradients. We therefore update reliability using the persistence of point tracks in $A_{j,k}$, without tracking line segments across frames. For each feature $i\in A_{j,k}$, let $\tau_{i}$ be its current tracking lifespan in frames. The number of supporting points and their accumulated lifespan are

(21) $$n_{j,k}=\left|A_{j,k}\right|,\;T_{j,k}^{\tau }=\sum_{i\in A_{j,k}}\tau_{i}.$$

The mean lifespan of the supporting tracks is

(22) $${\bar{\tau }}_{j,k}=\frac{T_{j,k}^{\tau }}{\max(n_{j,k},1)}.$$

To avoid bias from unsupported segments, the count scale is estimated only from segments with nonzero point support. Let

(23) $$J_{k}^{+}=\left\{j|n_{j,k}>0\right\},\;n_{90,k}^{+}=P_{90}\;\left({\left(n_{j,k}\right)}_{j\in J_{k}^{+}}\right),$$

where $P_{90}\left(\cdot \right)$ denotes the empirical 90th percentile over the indexed samples, preserving repeated support counts. If $J_{k}^{+}=\emptyset$, the support branch is inactive and all segments retain their image-evidence reliability. Otherwise, the count-based and lifespan-based support scores are computed as

(24) $$\begin{array}{cc} s_{\mathrm{cnt},j,k} & =\mathrm{clip}\;\left(\frac{n_{j,k}}{\max(n_{90,k}^{+},1)},\;0,\;1\right),\\ s_{\mathrm{life},j,k} & =\mathrm{clip}\;\left(\frac{{\bar{\tau }}_{j,k}}{\tau_{0}},\;0,\;1\right), \end{array}$$

where $\tau_{0}$ is a fixed lifespan scale.

When the support branch is active, the support-driven reliability is defined as

(25) $$q_{\mathrm{supp},j,k}=\frac{1}{2}\left(s_{\mathrm{cnt},j,k}+s_{\mathrm{life},j,k}\right).$$

The line reliability after support updating is obtained by

(26) $$\begin{array}{cc} q_{\mathrm{line},j,k} & =\left\{\begin{array}{cc} \alpha q_{\mathrm{img},j,k}+\left(1-\alpha \right)q_{\mathrm{supp},j,k},\; & n_{j,k}>0,\\ q_{\mathrm{img},j,k},\; & \mathrm{otherwise}, \end{array}\right. \end{array}$$

Thus, point-observation support strengthens a segment only when valid associated points exist; otherwise, the segment remains governed by image evidence. The fixed convex weight is kept within the unit interval, so $q_{\mathrm{line},j,k}\in \left[0,1\right]$ when its inputs are bounded.

#### 4.2.4. Directional Consistency as Soft Reliability Attenuation

In structured scenes, reliable line orientations often concentrate around a few dominant directions. We use this concentration as a soft prior and disable it when the previous-frame orientation distribution is weak.

Let

(27) $$D_{k-1}={\left\{\left(\theta_{r,k-1},q_{\mathrm{final},r,k-1}\right)\right\}}_{r\in J_{k-1}}$$

be the orientation–quality pairs of all line segments detected in the previous frame. This set is used as a distributional prior and does not imply one-to-one line correspondence across frames.

The interval [0,π) is divided into $B_{\theta }$ uniform bins:

(28) $$B_{b}=\left[\frac{(b-1)\pi }{B_{\theta }},\frac{b\pi }{B_{\theta }}\right),\;b=1,\ldots ,B_{\theta }.$$

For bin $B_{b}$, the accumulated quality weight is

(29) $$W_{b,k-1}=\sum_{r:\theta_{r,k-1}\in B_{b}}q_{\mathrm{final},r,k-1},\;W_{k-1}=\sum_{b=1}^{B_{\theta }}W_{b,k-1}.$$

A bin is considered valid only when the previous-frame orientation distribution contains enough prior segments and the bin has nontrivial relative support:

(30) $$V_{k-1}=\left\{b\;|\;|D_{k-1}|\geq N_{\theta },\;\frac{W_{b,k-1}}{\max(W_{k-1},\varepsilon_{\deg})}\geq W_{\min}\right\},$$

where $N_{\theta }$ is the minimum number of previous-frame line segments used to activate the directional prior. This gate keeps the directional prior active only under sufficient orientation support and prevents weak bins from defining principal directions.

For compact notation, define the folding operator:

(31) $${\mathrm{fold}}_{\pi }\left(\varphi \right)=\varphi -\pi \left\lfloor \frac{\varphi }{\pi }\right\rfloor ,\;{\mathrm{fold}}_{\pi }\left(\varphi \right)\in \left[0,\pi \right).$$

For each valid bin $b\in V_{k-1}$, the principal orientation is computed as the quality-weighted mean within that local orientation bin:

(32) $${\hat{\theta }}_{b,k-1}=\frac{\sum_{r:\theta_{r,k-1}\in B_{b}}q_{\mathrm{final},r,k-1}\theta_{r,k-1}}{\max\;\left(\sum_{r:\theta_{r,k-1}\in B_{b}}q_{\mathrm{final},r,k-1},\varepsilon_{\deg}\right)}.$$

The set of principal directions is

(33) $${\hat{\Theta }}_{k-1}=\left\{{\hat{\theta }}_{b,k-1}|b\in V_{k-1}\right\}.$$

If ${\hat{\Theta }}_{k-1}=\emptyset$, the directional prior is disabled.

We define the axial angular distance as

(34) $$d_{\pi }\left(\theta_{1},\theta_{2}\right)=\min\;\left(|\theta_{1}-\theta_{2}\left|,\pi -\right|\theta_{1}-\theta_{2}|\right),$$

which measures the shortest distance on the unoriented line-orientation manifold [0,π). For a current segment $L_{j,k}$, the nearest-principal-direction deviation is

(35) $$\Delta \theta_{j,k}=\min_{\hat{\theta }\in {\hat{\Theta }}_{k-1}}d_{\pi }\left(\theta_{j,k},\hat{\theta }\right).$$

The corresponding directional consistency score is

(36) $$s_{\mathrm{dir},j,k}=\exp\;\left[-\frac{1}{2}{\left(\frac{\Delta \theta_{j,k}}{\sigma_{\theta }}\right)}^{2}\right].$$

The attenuation width is fixed for all sequences and only controls how softly a segment is downweighted when it deviates from the dominant directions.

The final line reliability is defined as

(37) $$\begin{array}{cc} q_{\mathrm{final},j,k} & =\left\{\begin{array}{cc} \beta q_{\mathrm{line},j,k}+\left(1-\beta \right)s_{\mathrm{dir},j,k},\; & {\hat{\Theta }}_{k-1}\neq \emptyset ,\\ q_{\mathrm{line},j,k},\; & \mathrm{otherwise} \end{array}\right. \end{array}$$

This convex update softly blends local segment reliability with temporal directional consistency when a valid orientation prior exists. Since both inputs and the blending weight are bounded in the unit interval, $q_{\mathrm{final},j,k}\in \left[0,1\right]$.

### 4.3. Scene-Level Intervention Regulation

The reliability above is local to individual segments and associated points, whereas intervention strength should depend on frame-level structural support. We therefore introduce a scene-level confidence $w_{\mathrm{scene},k}$ to regulate front-end conditioning without modifying the back-end update.

A point is regarded as line-supported only when it is geometrically consistent with a reliable segment in the current frame and has accumulated sufficient track-level persistence. The set of good points is defined as

(38) $$\begin{array}{cc} G_{p,k}=\{i\in I_{k}^{\mathrm{assoc}}\;|\; & d_{i,k}\leq d_{\mathrm{good}},\\ & q_{\mathrm{final},j^{★}\left(i,k\right),k}\geq q_{\min},\\ & \tau_{i}\geq \tau_{\min}\}. \end{array}$$

The good-point ratio is

(39) $$r_{p,k}=\frac{|G_{p,k}|}{\max(|I_{k}^{\mathrm{raw}}|,1)}.$$

A line segment is regarded as active structural support if it is associated with at least one good point:

(40) $$G_{\ell ,k}=\left\{j\in J_{k}\;|\;\exists \;i\in G_{p,k}\;s.t.\;j^{★}\left(i,k\right)=j\right\}.$$

The good-line ratio is then

(41) $$r_{\ell ,k}=\frac{|G_{\ell ,k}|}{\max(|J_{k}|,1)}.$$

The raw scene score combines point-level and line-level structural support:

(42) $$s_{k}=\lambda_{p}r_{p,k}+\left(1-\lambda_{p}\right)r_{\ell ,k}.$$

The fixed weighting balances point-level support and line-level structural coverage. To suppress frame-to-frame fluctuations, an exponential moving average is applied:

(43) $${\bar{s}}_{k}=\eta {\bar{s}}_{k-1}+\left(1-\eta \right)s_{k},$$

with initialization ${\bar{s}}_{k_{0}}=s_{k_{0}}$ for the first processed frame $k_{0}$. The smoothing weight is fixed across all sequences. Finally, the scene-level line confidence is obtained by bounded linear mapping:

(44) $$w_{\mathrm{scene},k}=\mathrm{clip}\;\left(\frac{{\bar{s}}_{k}-s_{\min}}{\max(s_{\max}-s_{\min},\varepsilon_{\mathrm{norm}})},0,1\right).$$

Here, $s_{\min}$ and $s_{\max}$ are lower and upper scene-support gates. A larger $w_{\mathrm{scene},k}$ indicates stronger reliable line-supported point evidence and modulates the following conditioning and pruning rules.

### 4.4. Unified Bounded Conditioning and Sparse Safeguard

Given $q_{\mathrm{final},j,k}$ and $w_{\mathrm{scene},k}$, the front end applies a unified reliability-gated intervention. Bounded normal-direction conditioning is the primary operation, while pruning is activated only as a sparse safeguard for highly inconsistent long-lived tracks under strong structural evidence.

For an associated feature $i\in I_{k}^{\mathrm{assoc}}$, write $j_{i}^{★}=j^{★}\left(i,k\right)$ for compactness. For segment $L_{j_{i}^{★},k}$, the unit tangent and unit normal are defined as

(45) $$u_{j_{i}^{★},k}=\frac{v_{j_{i}^{★},k}}{∥v_{j_{i}^{★},k}{∥}_{2}},\;n_{j_{i}^{★},k}=\left[\begin{array}{c} -u_{y,j_{i}^{★},k}\\ u_{x,j_{i}^{★},k} \end{array}\right],$$

where $u_{j_{i}^{★},k}={\left[u_{x,j_{i}^{★},k},u_{y,j_{i}^{★},k}\right]}^{⊤}$. Using the projection point $\pi_{i,j_{i}^{★},k}$ defined in (16), the signed normal residual is

(46) $$e_{i,k}={\left(p_{0,k}^{i,\mathrm{raw}}-\pi_{i,j_{i}^{★},k}\right)}^{⊤}n_{j_{i}^{★},k}.$$

The magnitude $|e_{i,k}|$ equals the normal point–line distance for the selected segment, while the sign specifies the side of the segment normal on which the raw point lies.

The point-level intervention strength is determined by both segment reliability and scene confidence:

(47) $$\phi_{i,k}=w_{\mathrm{scene},k}\mathrm{clip}\;\left(\frac{q_{\mathrm{final},j_{i}^{★},k}-q_{0}}{\max(1-q_{0},\varepsilon_{\deg})},0,1\right),$$

where $\varepsilon_{\deg}>0$ prevents numerical degeneracy. This term does not enlarge the displacement cap; instead, it attenuates the soft update when the segment or scene evidence is weak.

The candidate is accepted for conditioning only when the raw normal residual falls inside the fixed displacement gate,

(48) $$|e_{i,k}|\;\leq \;\Delta_{0}.$$

For an accepted candidate, the robust distance weight and soft update factor are defined as

(49) $$r_{i,k}=\frac{1}{1+\left(\right|e_{i,k}{\left|/\sigma_{\mathrm{px}}\right)}^{2}},\;a_{i,k}=\mathrm{clip}\;\left(\gamma \phi_{i,k}r_{i,k},0,1\right),$$

where $\sigma_{\mathrm{px}}$ is the assumed pixel-noise scale and γ is the fixed soft-update coefficient.

The bounded normal-direction displacement is then defined as

(50) $$\delta_{i,k}=a_{i,k}\left(\pi_{i,j_{i}^{★},k}-p_{0,k}^{i,\mathrm{raw}}\right).$$

When $|e_{i,k}|>\Delta_{0}$, the point is left unchanged by the conditioning step. Otherwise, the soft update moves the point only part of the way toward the projected line position. Since $0\leq a_{i,k}\leq 1$ and the accepted candidate satisfies (48),

(51) $${∥\delta_{i,k}∥}_{2}\leq a_{i,k}\left|e_{i,k}\right|\leq \Delta_{0},$$

the primary-view perturbation is bounded. When $q_{\mathrm{final},j_{i}^{★},k}\leq q_{0}$ or $w_{\mathrm{scene},k}$ is close to zero, the soft update factor vanishes or becomes negligible. For the reported implementation, γ=0.5, $\sigma_{\mathrm{px}}=1$ pixel, $\Delta_{0}=0.6$ pixels, and $0\leq \phi_{i,k}\leq 1$. Hence, a tighter implementation-specific bound is

(52) $${∥\delta_{i,k}∥}_{2}\leq \max_{0\leq \left|e\right|\leq \Delta_{0}}\frac{\gamma |e|}{1+\left(|e\right|/\sigma_{\mathrm{px}}{)}^{2}}=\frac{0.5\times 0.6}{1+0.6^{2}}\approx 0.221\;\mathrm{pixels}.$$

The pruning safeguard removes a track only when reliable structural support and persistent point–line inconsistency coexist. The set of pruned tracks is

(53) $$\begin{array}{cc} R_{k}=\{i\in I_{k}^{\mathrm{assoc}}\;|\; & w_{\mathrm{scene},k}>w_{\mathrm{prune}},\\ & w_{\mathrm{scene},k}q_{\mathrm{final},j_{i}^{★},k}\geq q_{\mathrm{prune}},\\ & \tau_{i}\geq \tau_{\mathrm{prune}},\\ & |e_{i,k}|>d_{\mathrm{prune}}\}. \end{array}$$

The pruning gate requires high scene confidence, effective segment reliability, track persistence, and point–line inconsistency simultaneously. The residual gates follow

(54) $$d_{\mathrm{good}}\leq d_{\mathrm{prune}}\leq d_{\mathrm{assoc}},$$

so pruning is triggered only by residuals that are larger than the good-support bound while still inside the association range. Features outside $R_{k}$ follow a retain-first policy:

(55) $${\tilde{I}}_{k}=I_{k}^{\mathrm{raw}}\setminus R_{k}.$$

For each retained feature, the primary-view coordinate passed to measurement packaging is

(56) $${\tilde{p}}_{0,k}^{i}=\left\{\begin{array}{cc} p_{0,k}^{i,\mathrm{raw}}+\delta_{i,k},\; & i\in {\tilde{I}}_{k}\cap I_{k}^{\mathrm{assoc}},\\ p_{0,k}^{i,\mathrm{raw}},\; & i\in {\tilde{I}}_{k}\setminus I_{k}^{\mathrm{assoc}}. \end{array}\right.$$

Thus, unassociated or weakly supported features are retained with baseline or near-baseline coordinates, whereas high-confidence associated points receive bounded normal-direction conditioning.

After conditioning and pruning, the retained feature set and conditioned primary-view coordinates are passed to the stereo point-measurement interface in Section 4.1.

### 4.5. Conservative Design Properties

The layer is a constrained measurement-conditioning rule rather than a replacement for the VIO measurement model. Its constants are fixed once according to tolerance ordering, reliability monotonicity, and conservative pruning requirements; the same setting is used for all public and private sequences. The key properties are therefore stated through inequalities and gating relations.

First, the intervention is interface-preserving. For every retained feature, the conditioned observation remains a four-dimensional stereo point measurement; no line state, line residual, or additional Kalman update is introduced.

Second, the visual-measurement perturbation is uniformly bounded. From (48)–(51),

(57) $$0\leq \phi_{i,k}\leq 1,\;0\leq a_{i,k}\leq 1,\;{∥\delta_{i,k}∥}_{2}\leq \Delta_{0}.$$

Thus, a point is conditioned only when its raw point–line residual lies within the prescribed displacement gate, and the accepted correction is a partial update rather than an unconditional projection onto the line. In the reported implementation, the tighter bound in (52) further limits the actual final coordinate update. This protects stereo measurement integrity when the right-camera coordinate is unchanged.

Third, line guidance is monotone with respect to reliability and scene confidence. If segment reliability or scene-level support becomes weak, $\phi_{i,k}$ decreases toward zero. Reliable line evidence can increase the partial-update factor within the fixed displacement gate, but cannot remove the upper bound in (57).

Fourth, pruning follows a retain-first policy. The distance gates obey

(58) $$d_{\mathrm{good}}\leq d_{\mathrm{prune}}\leq d_{\mathrm{assoc}},$$

and the pruning reliability gates are placed in the high-confidence regime, i.e.,

(59) $$q_{\mathrm{prune}}\geq q_{\min}\geq q_{0},\;w_{\mathrm{prune}}>0.$$

Consequently, weakly supported features are retained with baseline or near-baseline coordinates. Pruning occurs only when scene confidence, segment reliability, track persistence, and point–line inconsistency are simultaneously high. The empirical pruned-point ratios in Section 5.2 verify that this safeguard remains sparse.

These properties make the module a conservative visual measurement-conditioning layer: structural lines regulate point observations before fusion, while the estimator state, stereo measurement dimension, and update interface remain unchanged. Section 5.2.5 further reports candidate-level stereo-consistency diagnostics for the primary-view intervention.

## 5. Experiments

### 5.1. Experimental Setup

Experiments were conducted on the public EuRoC MAV dataset [33] and a private outdoor robotic measurement dataset. EuRoC is used as the primary reproducible benchmark because it provides synchronized stereo images, inertial measurements, calibrated sensor parameters, and ground-truth trajectories for indoor micro-aerial-vehicle motion. It is challenging for visual–inertial odometry due to rapid camera motion, motion blur, illumination variation, texture-poor regions, repetitive structures, and different levels of visual and inertial excitation. In particular, the machine-hall sequences contain long structural lines, industrial clutter, and larger-scale motion, whereas the Vicon-room sequences involve shorter-range motion and less persistent structural support. These properties make EuRoC suitable for evaluating whether line-aware measurement conditioning benefits structured scenes without degrading performance in less favorable ones.

The private dataset complements EuRoC with real-world ground-robot measurements. The private robot sequences were collected in an outdoor campus environment in Harbin, China. They cover road, corridor, vegetation-side, square-loop, and open-area routes under varying illumination, texture richness, and structural sparsity. The evaluated route lengths are 466.25 m, 565.25 m, and 380.88 m for Robot1, Robot2, and Robot3, respectively. Unlike EuRoC, these sequences include outdoor line responses from road edges, fences, curbs, building boundaries, shadows, and vegetation, which test whether the proposed scene-adaptive confidence can distinguish persistent structural support from unstable visual line cues. The private sequences were captured using an Intel RealSense D435i stereo camera (Intel Corporation, Santa Clara, CA, USA) and its built-in IMU as the VIO input, while a CGI-1010 inertial navigation module (Shanghai Huace Navigation Technology Ltd., Shanghai, China) aided by RTK GNSS provides the independent reference position and heading. According to the manufacturer specification, the CGI-1010 provides RTK positioning accuracy of ≤2 cm + 1 ppm (1σ), attitude accuracy of 0.01° (1σ), and heading accuracy of 0.05° under single-antenna dynamic alignment. All sensor streams were collected through ROS2. The stereo intrinsic parameters and the camera–IMU extrinsic calibration of the D435i were estimated using Kalibr before data collection and then kept fixed for all private sequences. The RTK-aided inertial reference was time-aligned to the VIO output using the recorded ROS2 timestamps and evaluated over the common valid interval after initialization. The main sensor configuration is summarized in Table 4. The evaluation therefore combines a controlled public benchmark with standard ground truth and deployment-oriented robot measurements with an independent navigation reference.

The main EuRoC comparison includes S-MSCKF, VINS-Mono, ROVIO, and the proposed full method. S-MSCKF is the unchanged stereo MSCKF baseline and provides the controlled reference for evaluating the effect of the proposed front-end measurement-conditioning layer under the same filtering back end. VINS-Mono and ROVIO are reported as external reference systems, representing optimization-based and filter-based VIO pipelines, respectively.

We also report a separate component ablation for the proposed front-end layer. The ablation variants are evaluated within the same additional ablation-run batch and are used to examine the roles of line-quality weighting, directional consistency, scene-level confidence, bounded point conditioning, and sparse pruning. In the proposed front end, line quality, point–line association, support-driven reliability, and directional consistency jointly provide a reliability estimate for safe point-measurement conditioning. Bounded conditioning is the dominant operation, whereas pruning is activated only as a sparse safeguard under strong structural evidence. Mechanism statistics, including scene-confidence activation, conditioning ratio, and pruned-point ratio, are reported separately to characterize this intervention.

S-MSCKF and the proposed method use the same stereo calibration, image resolution, and front-end tracking settings. VINS-Mono and ROVIO are run with their public EuRoC configurations and without sequence-specific tuning.

The proposed line-aware front end uses one fixed parameter setting for all EuRoC and private robot sequences. The parameters were selected according to the bounded-intervention design considerations described in Section 4.5 and then kept fixed throughout all the reported experiments. The fixed settings in Table 5 follow these design criteria. The displacement bound is tied to the assumed image-measurement noise level, pruning follows the ordered residual gates $d_{\mathrm{good}}\leq d_{\mathrm{prune}}\leq d_{\mathrm{assoc}}$ and is capped by a small maximum removal ratio, and the scene-confidence term is temporally smoothed to suppress switching caused by transient line detections. Potential adverse behavior may arise when the displacement bound or scene-confidence gate becomes overly permissive, consistent with the weak-parallax boundary case discussed below.

The FLD configuration is fixed throughout all the reported experiments and is listed explicitly in Table 5. The minimum-length threshold controls a practical trade-off: a smaller value may admit more short or transient segments, whereas a larger value may suppress useful structural support. In the present study, the detector configuration is treated as a fixed front-end implementation choice, while the proposed contribution focuses on the subsequent reliability estimation, scene-adaptive regulation, and bounded measurement conditioning. A systematic detector-level sensitivity study is left for future work.

Point–line VIO/SLAM systems such as PL-VIO and PL-VINS explicitly introduce line features into the estimator or mapping back end through line states, line reprojection residuals, or graph-based structural constraints. They are therefore discussed as related point–line modeling approaches rather than used as component-wise baselines for the proposed back-end-preserving front-end conditioning layer. Direct system-level comparisons with such methods can be informative, but they also mix several factors, including feature representation, line residual modeling, optimization or filtering strategy, and implementation-specific marginalization. The primary controlled comparison is therefore between S-MSCKF and its line-aware variants, which share the same state representation, stereo point-measurement interface, and MSCKF update formulation. This comparison isolates the front-end measurement-conditioning effect under the evaluated S-MSCKF back end, rather than comparing complete VIO systems with different line-measurement formulations.

On EuRoC, we report the root-mean-square error of the absolute trajectory error (ATE RMSE) and the absolute rotation error (ARE RMSE). ARE is computed as the geodesic rotation error on SO(3) and reported in degrees. Both metrics are computed using EVO [34] after SE(3) alignment, with the same alignment protocol for all methods. S-MSCKF and the proposed line-aware variants are run five times per sequence, and the mean result is reported together with run-to-run variability statistics. VINS-Mono and ROVIO are included as external reference systems using their public EuRoC configurations. The table should be interpreted as a mean-performance comparison rather than a statistical significance test. The reported EuRoC comparison includes MH01–MH05, V1_01–V1_03, and V2_01–V2_02. In the original S-MSCKF evaluation, Sun et al. report that their method does not work properly on V2_03_difficult because KLT optical flow is used for both temporal feature tracking and stereo matching for efficiency [9]. The continuous brightness inconsistency between the stereo images causes stereo feature-matching failures and filter divergence [9]. For consistency with this evaluation setting, V2_03_difficult is not included in the averaged results. For the outdoor dataset, we first report 2-D Sim(2)-aligned XY absolute pose error RMSE (XY APE RMSE) and yaw RMSE. Because the outdoor VIO and RTK-aided INS trajectories are produced in independent local frames, this metric emphasizes planar trajectory-shape and heading consistency over the common valid interval. We additionally report a scale-preserving SE(2) diagnostic, where only planar rotation and translation are estimated and the scale is fixed to one. The corresponding Sim(2) scale factor is also listed to indicate how much scale correction is introduced by the similarity alignment. All methods are evaluated over their common valid interval after initialization, and failed runs are marked explicitly. Per-sequence mean ± standard deviation values for the five independent controlled EuRoC runs are reported in Table A1. The average and maximum within-sequence variability are summarized after the main EuRoC comparison.

In addition to trajectory-error metrics, we report diagnostic statistics characterizing scene-confidence activation, conditioning frequency, sparse pruning, feature-track persistence, and candidate-level stereo geometry. The diagnostic runs are reported on four representative EuRoC sequences covering line-rich machine-hall motion and more challenging Vicon-room cases. The candidate-level stereo diagnostics characterize the geometric risk of line-supported adjustment proposals, whereas the final coordinate update is governed by the bounded soft-conditioning rule in Section 4.4. These diagnostics characterize final pose behavior, candidate-level stereo geometry, and the analytical boundedness of the conditioning layer without conflating candidate-level quantities with the final coordinate update.

### 5.2. Public Benchmark Evaluation

#### 5.2.1. Overall Accuracy on EuRoC

Table 6 reports the quantitative results on the ten reported EuRoC sequences. At the precision reported in Table 6, OURS obtains lower or equal ATE RMSE on all ten sequences. The more precise five-run statistics in Table A1 reveal a small increase on V1_02, which is examined separately as a boundary case in Section 5.2.6. On the machine-hall sequences MH01–MH05, the relative ATE RMSE reductions are approximately 3–27%. ARE RMSE reductions are observed on all the reported EuRoC sequences except V1_02. Over the reported sequences, the sequence-averaged ATE RMSE decreases from 0.184 m to 0.160 m, and the sequence-averaged ARE RMSE decreases from 1.78° to 1.56°.

Table 7 summarizes the run-to-run variability of the two controlled S-MSCKF-based methods. The within-sequence standard deviation is computed from five independent runs for each reported EuRoC sequence and then averaged over the ten sequences. OURS has a slightly lower average ATE variability and a lower average ARE variability than S-MSCKF, although the largest ARE variation remains on V1_03. The results therefore support the mean-error comparison in Table 6, while also indicating that the reported improvement should be interpreted together with sequence-dependent variability.

The improvements are most evident in sequences where stable straight structures persist across frames and repeatedly provide reliable geometric support for point-measurement conditioning. On V1_02, the reported ATE RMSE is unchanged at the precision shown in Table 6, while the more precise five-run statistics in Table A1 indicate a small increase. The ARE RMSE also increases slightly, and this sequence is analyzed as a boundary case in Section 5.2.6. The overall results indicate that the proposed front-end layer tends to improve structured scenes while keeping the unchanged S-MSCKF update interface.

#### 5.2.2. Component Ablation

Table 8 reports an additional component ablation of the proposed front-end layer under the EuRoC evaluation protocol. It compares the full method with five internal variants that remove or emphasize different parts of the reliability estimation and intervention rule. The full method uses line-quality weighting, directional consistency, scene-level confidence, bounded normal-direction conditioning, and sparse pruning together. The *w/o line-quality weighting* variant removes the image-level quality weight from the reliability score, and the *w/o directional consistency* variant removes the temporal orientation-consistency term. The *w/o scene confidence* variant removes scene-level attenuation and therefore applies line-aware intervention whenever the local reliability gates are satisfied. The *conditioning-focused* variant keeps bounded point conditioning as the dominant operation and disables the sparse pruning safeguard. The *pruning-focused* variant keeps the selective removal of strongly inconsistent line-supported tracks but disables coordinate conditioning. These variants isolate the main roles of reliable line selection, scene-level regulation, bounded coordinate adjustment, and feature removal.

The ablation results show that the full method provides the largest averaged reduction in both ATE and ARE. Removing line-quality weighting causes the largest degradation, with the averaged ATE becoming slightly worse than the S-MSCKF baseline. This result indicates that the proposed module relies on reliable line evidence rather than on geometric proximity to detected segments alone. Removing directional consistency also reduces both translational and rotational gains, suggesting that temporally stable line orientations help suppress transient or weakly supported line responses. Disabling scene-level confidence preserves most of the translational improvement but reduces the ARE gain from 12.4% to 4.5%, indicating that scene-level attenuation is mainly useful for suppressing unreliable intervention that affects rotation consistency. The conditioning-focused variant remains closer to the full method than the pruning-focused variant in translational error, which supports the design choice that bounded point-measurement conditioning is the primary operation, whereas pruning should remain a sparse safeguard rather than the main source of improvement. Individual sequences can still favor a simpler variant when the detected line support is sparse, distant, or weakly persistent. The full method is therefore designed for averaged robustness under mixed scene conditions rather than for sequence-specific optimality.

#### 5.2.3. Sensitivity to the Residual Conditioning Bound

We further examine the sensitivity of the residual conditioning bound $\Delta_{0}$, which controls the maximum raw normal residual eligible for line-aware conditioning. The actual final coordinate update is further scaled by the reliability term and robust distance weight in (49) and (50). The tested values are 0.4, 0.6, and 0.8 pixels. The default value $\Delta_{0}=0.6$ pixels is the fixed setting used in the main experiments, and the other two settings are evaluated only to assess the sensitivity of the bound around the nominal setting.

Table 9 shows that the nominal bound yields the lowest sequence-averaged ATE and ARE among the tested settings. A smaller bound can underuse reliable line support, whereas a larger bound can admit more imperfect point–line associations into the conditioning step. These results therefore support the fixed moderate residual-admission bound used in the main experiments.

#### 5.2.4. Qualitative and Mechanism Diagnostics

**Figure 2:**
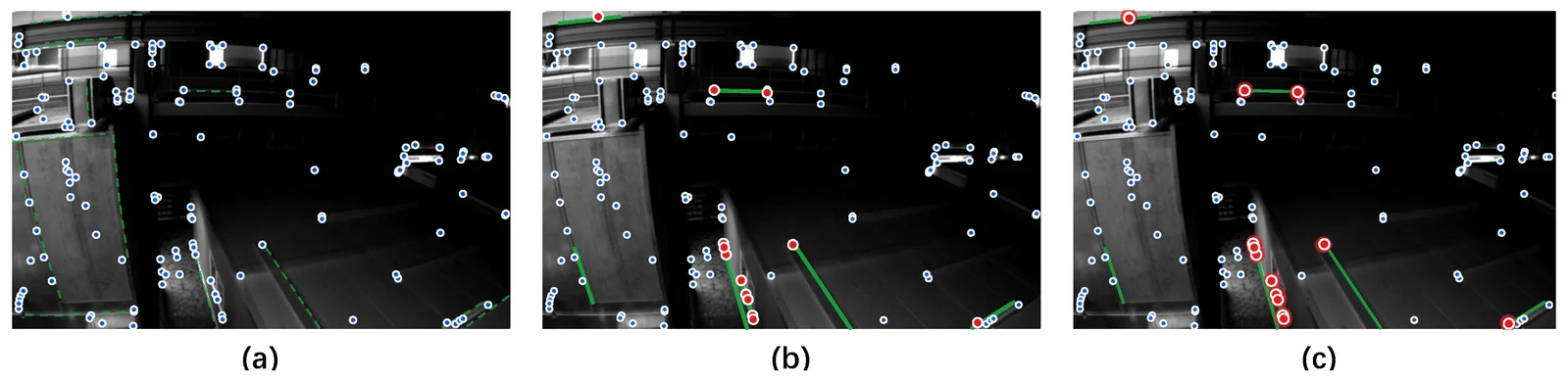
Image-level behavior of the proposed line-aware point conditioning on the EuRoC MH04 sequence. (**a**) Raw front-end detections, where blue points denote grid-distributed point features and green dashed segments denote candidate image lines. (**b**) Line-support association, where red points denote raw point features supported by reliable line segments and green solid segments denote reliable line structures. (**c**) Conditioned output, where red points denote the conditioned locations of line-supported points and light hollow markers indicate their original raw positions, illustrating the bounded image-domain displacement.

Figure 2 illustrates the image-level behavior of the line-aware point conditioning on EuRoC MH04. Figure 2a shows the raw front-end point features and candidate image-line segments extracted by the line-aware module. In Figure 2b, reliability scoring and point–line association identify point features supported by reliable structural lines. In Figure 2c, these line-supported points are locally adjusted to form conditioned point measurements, while ordinary point features are retained. The visualization shows how reliable structural cues in machine-hall scenes are converted into bounded point-measurement conditioning before the measurements are passed to the evaluated S-MSCKF update.

**Figure 3:**
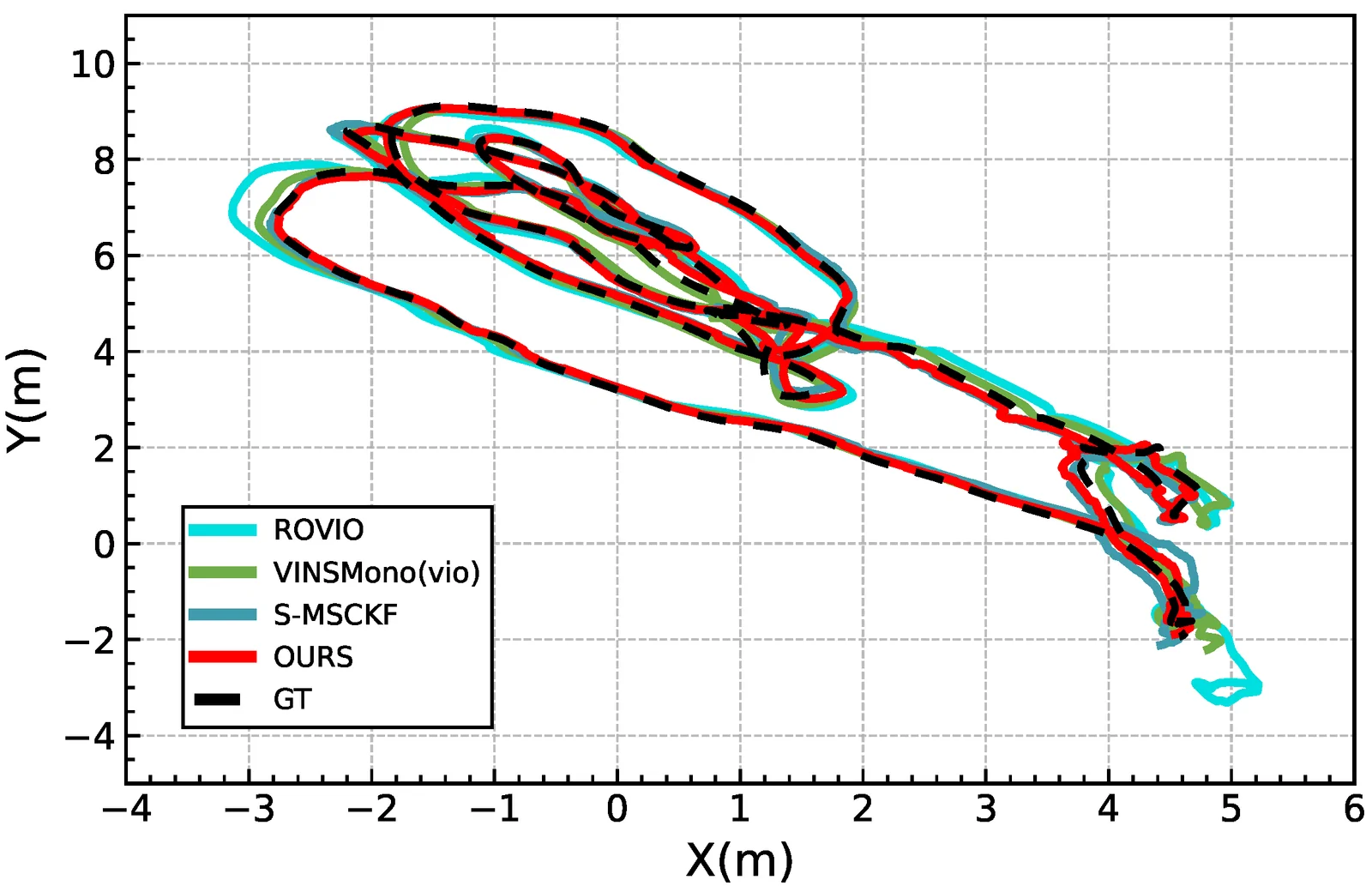
Aligned XY-plane trajectories on the EuRoC MH01 sequence.

**Figure 4:**
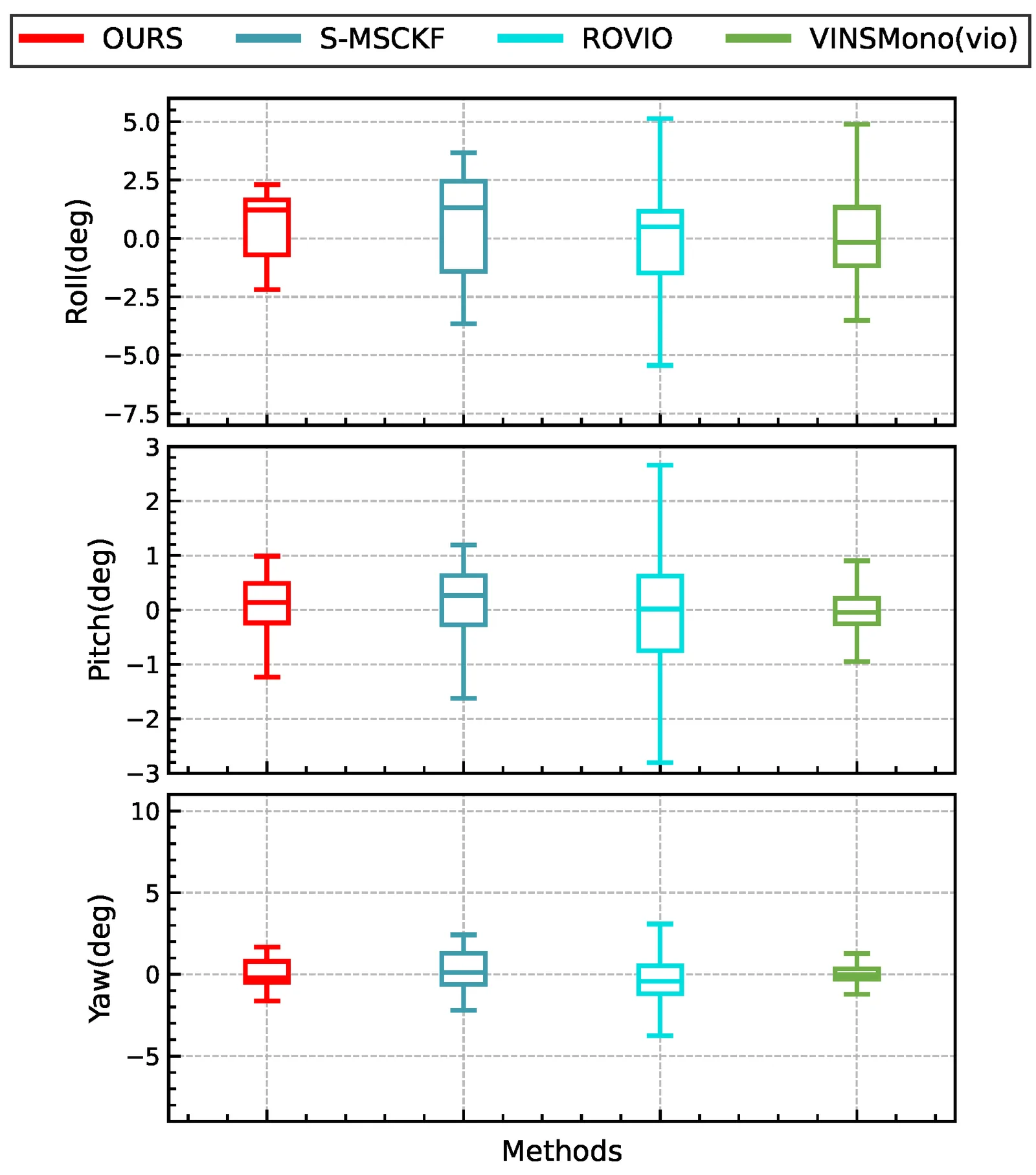
RPY error boxplots on the EuRoC MH01 sequence.

Consistent with the quantitative results, Figure 3 shows the reduced trajectory drift of OURS on MH01, especially around turns. The RPY error boxplots in Figure 4 further show improved yaw stability, while roll and pitch remain comparable to the baseline. These plots indicate that the proposed front end improves trajectory consistency mainly by regularizing structurally supported point observations rather than by changing the back-end update formulation.

Table 10 reports the behavior of the line-aware front-end intervention on representative diagnostic sequences. Line guidance is activated on most frames, and about 39–59% of line-associated front-end feature candidates receive conditioning. In contrast, the pruned-point ratio remains below 1% on all reported sequences. These statistics show that the intervention is dominated by reliability-gated conditioning rather than aggressive feature deletion. Together, these diagnostics support treating the conditioning–safeguard rule as a conservative intervention, with scene-adaptive confidence controlling when the intervention is strengthened or attenuated.

**Table 10:** Front-end behavior statistics of the proposed line-aware intervention.

Seq.	Mean $w_{\mathrm{scene}}$	Active Frames	Cond. Ratio	Pruned Ratio
MH01	0.666	95.3%	54.7%	0.41%
MH04	0.726	99.6%	47.8%	0.66%
V1_02	0.706	99.0%	39.3%	0.89%
V2_01	0.882	99.9%	58.8%	0.92%

*Note:* Statistics are computed from the same diagnostic batch as Table 11. Active frames denote frames with nonzero scene-level line guidance. Cond. ratio denotes the fraction of line-associated front-end feature candidates receiving conditioning. Pruned ratio denotes the fraction of tracked front-end points removed by the sparse pruning safeguard.

**Table 11:** Candidate-level stereo-consistency diagnostics of the proposed line-aware conditioning. All perturbation values are measured in pixels.

Seq.	# Cand.	Mean $|\Delta u_{\mathrm{cand}}|$	P95 $|\Delta u_{\mathrm{cand}}|$	Mean $|\Delta v_{\mathrm{cand}}|$	P95 $|\Delta v_{\mathrm{cand}}|$	Mean Signed $\Delta e_{v}$
MH01	13,978	0.095	0.440	0.143	0.696	0.0035
MH04	7821	0.090	0.438	0.185	0.818	−0.0076
V1_02	7582	0.184	0.791	0.133	0.723	−0.0053
V2_01	17,662	0.166	0.771	0.084	0.420	0.0021

*Note:* The perturbation statistics are computed point-wise over line-associated adjustment proposals from an auxiliary diagnostic stream. This diagnostic population is evaluated independently of the conditioning admission rule in (48) and is used only to characterize candidate-level stereo geometry. For rectified stereo measurements, $|\Delta u_{\mathrm{cand}}|$ corresponds to the candidate disparity perturbation, whereas $|\Delta v_{\mathrm{cand}}|$ corresponds to the candidate vertical-coordinate perturbation of the left measurement. These diagnostic quantities should not be interpreted as the actual final coordinate displacement passed to the back end. The final primary-view update in the reported method is governed by (49) and (50) and satisfies the implementation-specific analytical bound in (52). The right-camera measurement remains the raw stereo-matching output. Here, $\Delta e_{v}=e_{v}^{\mathrm{diag}}-e_{v}^{\mathrm{raw}}$ is a signed quantity and is reported only to diagnose systematic vertical-stereo bias.

#### 5.2.5. Candidate-Level Stereo-Consistency Diagnostics

Since the proposed line-aware conditioning acts on the primary-view measurement while keeping the right-camera correspondence unchanged, we further report a candidate-level stereo-consistency diagnostic. In the rectified stereo front end, the right-camera coordinate remains the direct stereo-matching measurement. An auxiliary diagnostic stream records the horizontal and vertical components of line-associated adjustment proposals, denoted by $|\Delta u_{\mathrm{cand}}|$ and $|\Delta v_{\mathrm{cand}}|$, respectively. This diagnostic population is evaluated independently of the conditioning admission rule in (48) and is used only to characterize candidate-level stereo geometry. These quantities are not the final reliability-weighted coordinate updates passed to the back end. We also compare the vertical-stereo mismatch before and after the recorded diagnostic proposal:

(60) $$e_{v}^{\mathrm{raw}}=\left|v_{l}^{\mathrm{raw}}-v_{r}^{\mathrm{raw}}\right|,\;e_{v}^{\mathrm{diag}}=\left|v_{l}^{\mathrm{diag}}-v_{r}^{\mathrm{raw}}\right|,$$

where the right-camera measurement remains the raw stereo-matching output. The signed change in vertical mismatch is defined as

(61) $$\Delta e_{v}=e_{v}^{\mathrm{diag}}-e_{v}^{\mathrm{raw}}.$$

The diagnostic proposal does not enforce unchanged epipolar residuals for every primary-view candidate. The vertical component is therefore quantified explicitly and is used to check whether the recorded diagnostic proposals produce a systematic vertical-stereo bias.

Table 11 reports the candidate-level stereo-consistency diagnostics associated with primary-view conditioning. Across the evaluated sequences, the reported mean horizontal candidate perturbation is below 0.19 pixels, and the reported 95th percentile is below 0.80 pixels. The reported vertical candidate perturbation is also subpixel at the mean and 95th-percentile levels, with the mean below 0.19 pixels and the 95th percentile below 0.82 pixels. Moreover, the mean signed change in vertical-stereo mismatch remains close to zero, ranging from −0.0076 to 0.0035 pixels. These statistics indicate that the recorded primary-view proposals do not exhibit a systematic vertical-stereo bias. They also clarify the role of the unchanged right-camera measurement: the method preserves the original stereo match and uses line evidence only to regularize the primary-view coordinate within the fixed stereo point-measurement interface. The diagnostic values therefore complement the analytical final-update bound in (52) rather than replacing it.

Using the implementation-specific final-update bound $|\delta_{u,i,k}|\leq 0.221$ pixels, the first-order relation in (5) gives the analytical approximation $\left|\Delta Z|/Z\leq 0.221/\right|d_{k}^{i,\mathrm{raw}}|$. For disparities of 5, 10, 20, and 40 pixels, this corresponds to approximate upper bounds of 4.42%, 2.21%, 1.11%, and 0.55%, respectively. This calculation is an analytical interpretation of the final bounded update, not an empirical depth-error measurement, and it is not obtained by substituting the candidate-level $\Delta u_{\mathrm{cand}}$ values in Table 11. It also explains why small-disparity and weak-parallax observations are more sensitive to primary-view conditioning.

#### 5.2.6. Failure-Case Analysis on V1_02

On V1_02, S-MSCKF and OURS have the same reported ATE RMSE of 0.12 m at the precision shown in Table 6. The more precise five-run statistics in Table A1 show a small increase from 0.116±0.004 m to 0.121±0.018 m for OURS. The ARE RMSE also increases slightly. We therefore examine this sequence as a boundary case below. This sequence contains weaker and less persistent line structures under more aggressive motion, and many detected segments correspond to distant background edges with limited parallax. Under such conditions, 2-D image-line support can appear structurally consistent in the image plane while providing limited multi-view geometric constraint. As a result, the scene-level confidence may become locally over-optimistic and activate line-aware conditioning in regions where the associated point tracks do not provide sufficiently persistent MSCKF constraints. This suggests that the current confidence model mainly captures image-domain structural support and does not explicitly distinguish nearby geometrically useful lines from distant weak-parallax line responses.

**Figure 5:**
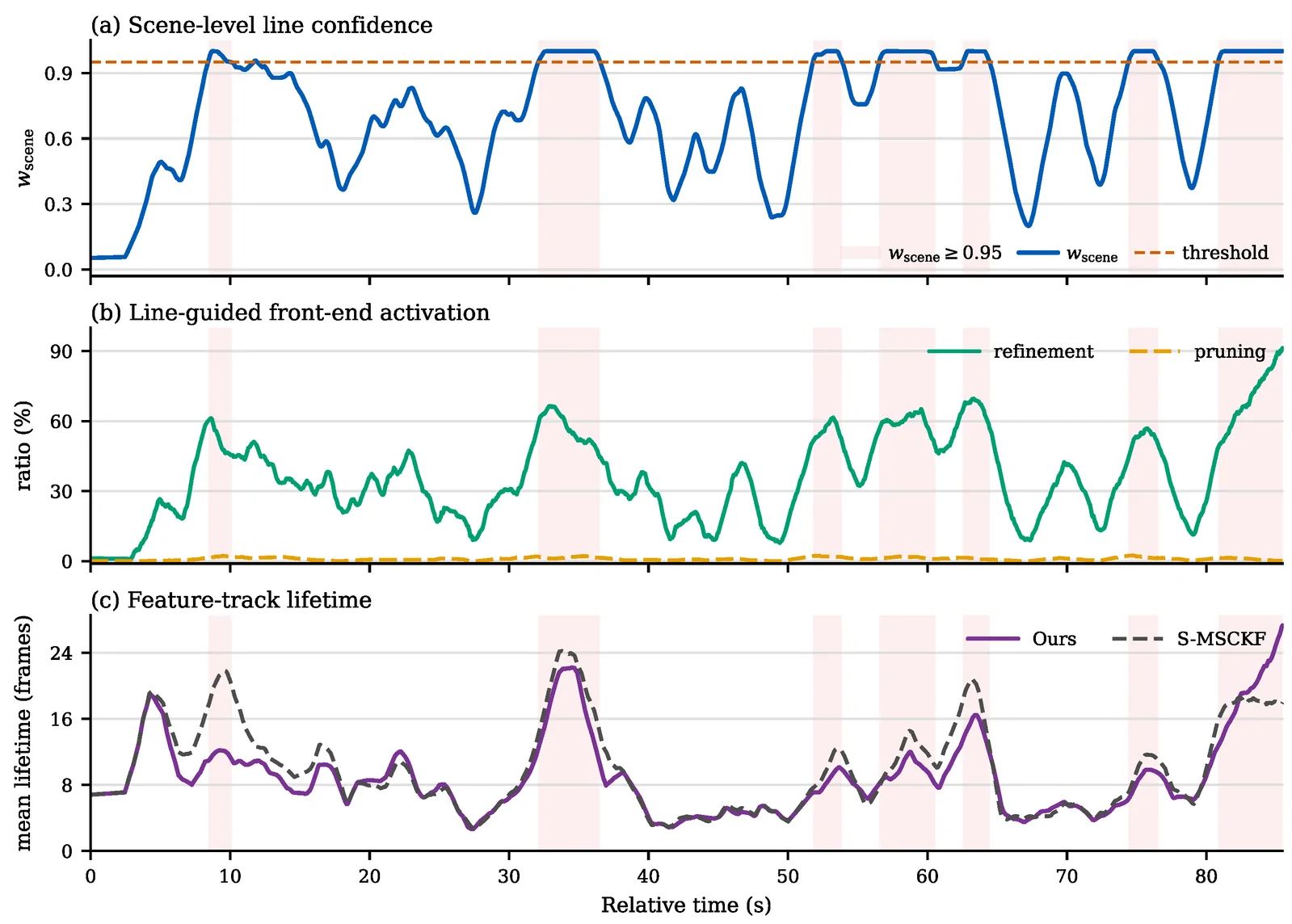
Temporal diagnostics on V1_02. Shaded regions denote frames where $w_{\mathrm{scene}}\geq 0.95$. The line-aware front end activates bounded conditioning more frequently in these intervals, while the pruned-point ratio remains low. The track-lifetime comparison indicates that the degradation is mainly related to reduced track persistence rather than increased measurement residuals.

Figure 5 and Table 12 provide a detailed diagnostic analysis. The degradation is not caused by worse instantaneous measurement quality: the line-aware front end slightly reduces the residual RMSE and residual P95 compared with S-MSCKF while maintaining a comparable gating pass ratio. The spatial distribution of features is also preserved, as indicated by nearly identical grid occupancy entropy and empty-grid ratio.

The main difference lies in feature-track persistence. The mean track lifetime decreases from 10.04 to 9.03, and the P90 lifetime decreases from 23.76 to 21.64. Meanwhile, the pruned-point ratio stays below 1%, showing that the degradation is not caused by aggressive feature pruning. A plausible explanation is that bounded conditioning improves local point–line consistency but slightly shortens track persistence in this sequence. Since MSCKF benefits from long-baseline multi-state point constraints, shortening a subset of long tracks may weaken the effective multi-view constraint even when instantaneous residual statistics improve.

The EuRoC results show that the line-quality scoring and scene-adaptive mechanism improve MSCKF accuracy in line-rich indoor environments, while maintaining baseline-level performance when reliable line support is limited. The V1_02 diagnostics further reveal a boundary case: overly confident 2-D line support from distant or weak-parallax structures can reduce track persistence, suggesting that future confidence models may benefit from explicit parallax- or depth-aware reliability terms.

### 5.3. Real-World Robot Measurement Experiment

**Figure 6:**
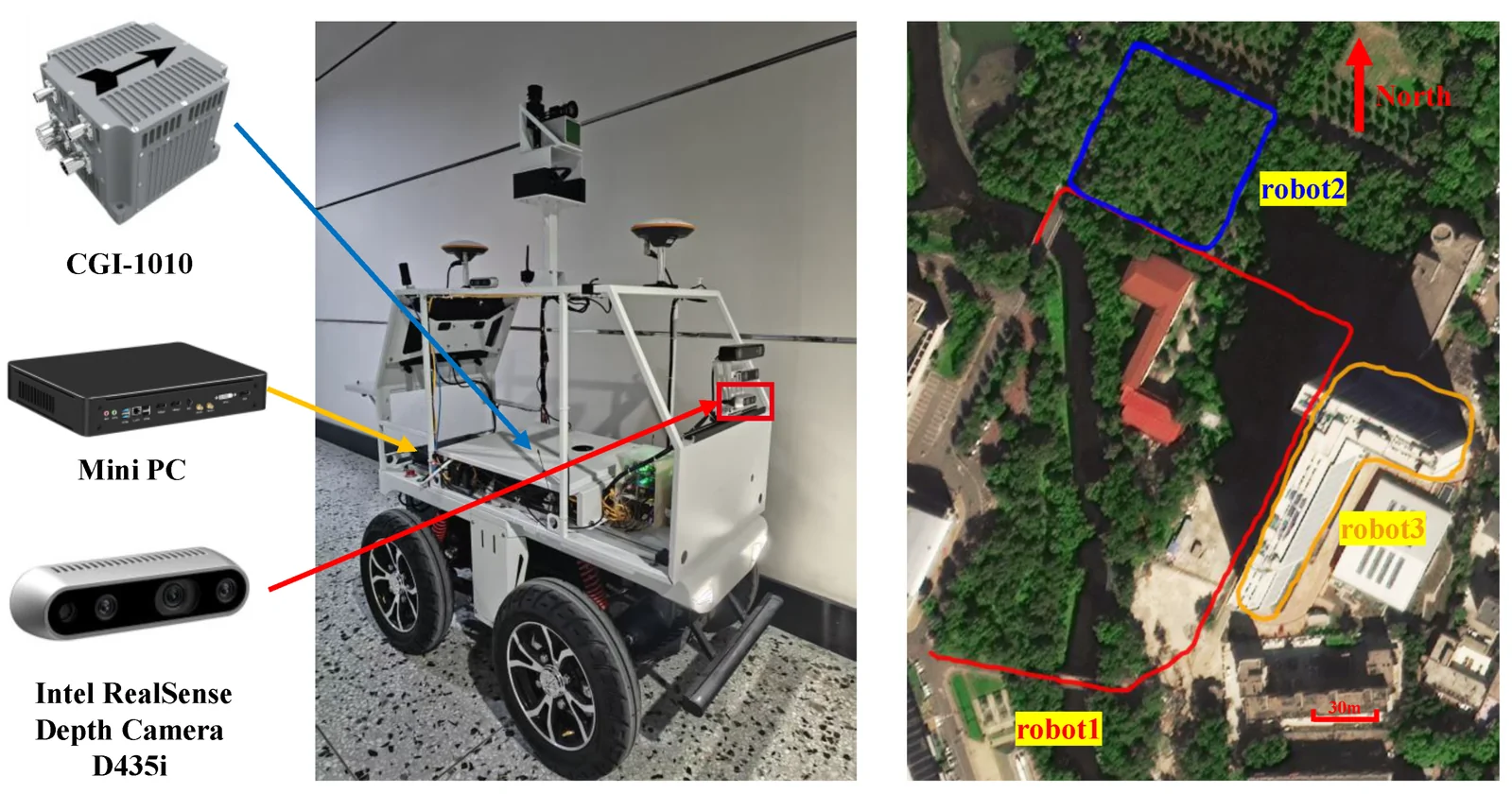
Overview of the ground robot platform and data-collection trajectories.

To complement the public benchmark with outdoor robot experiments, we evaluate the line-aware front end on three private sequences, denoted as *Robot1*, *Robot2*, and *Robot3*. These sequences cover long straight-line driving, square-loop motion, turning maneuvers, and open-area motion under outdoor illumination changes and structural sparsity. The ground robot platform and the corresponding data-collection trajectories are shown in Figure 6.

Table 13 summarizes the main characteristics of the three private robot sequences. The route lengths are computed from the RTK-aided reference trajectories, and the durations are obtained from the recorded CGI-1010 timestamps.

**Figure 7:**
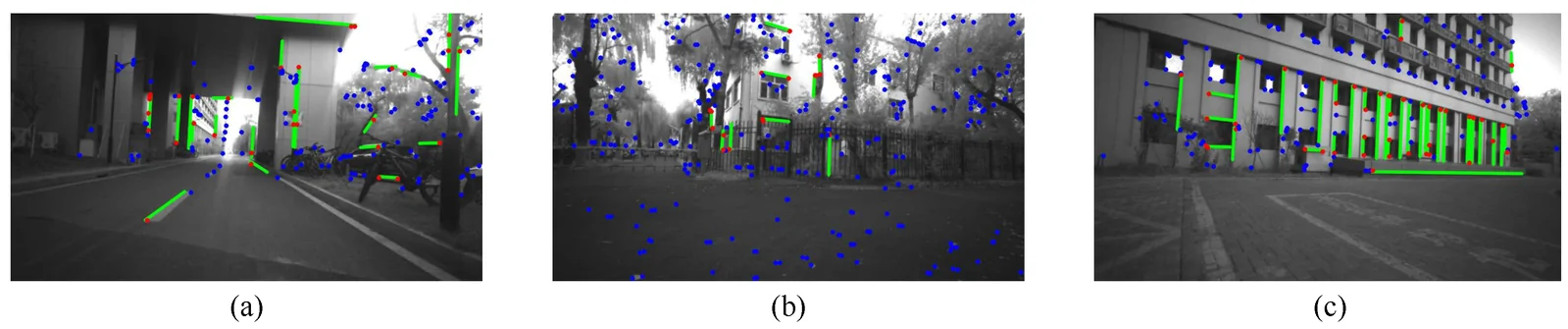
Point–line association examples on the private sequences: (**a**) Robot1, (**b**) Robot2, and (**c**) Robot3. Green lines denote detected candidate line segments, blue dots denote tracked point features, and red dots denote point features associated with the selected line support.

Representative point–line association results from the private dataset are visualized in Figure 7. Detected candidate line segments are shown in green. The examples show that associated points are mainly distributed around persistent structural boundaries, such as road edges and fences, whereas line responses caused by foliage, shadows, or weakly persistent texture are less likely to form stable point–line support. This qualitative behavior is consistent with the line-aware front end, which uses line evidence as a bounded image-domain prior rather than as a hard geometric constraint.

Quantitative results on *Robot1*–*Robot3* are summarized in Table 14.

To examine whether the outdoor comparison depends on similarity-scale correction, Table 15 reports a scale-preserving SE(2) diagnostic using the same trajectory batch. The yaw RMSE is the heading metric in Table 14, which is independent of the XY scale factor. The additional diagnostic shows that the Sim(2) alignment applies noticeable scale correction on the outdoor sequences, especially on Robot1 and Robot3. Therefore, the Sim(2)-aligned XY errors in Table 14 should be interpreted primarily as planar trajectory-shape consistency rather than as direct evidence of metric-scale accuracy. Under SE(2) alignment, the line-aware variants remain competitive on Robot2, but the full method does not uniformly reduce XY error on Robot1 and Robot3. This result is consistent with the boundary-case analysis: line-aware conditioning can improve local structural consistency, while the SE(2) diagnostic reveals residual metric-scale inconsistency in the outdoor trajectories. Potential contributions from synchronization and reference-frame calibration cannot be isolated by the present experiment.

The route lengths provide scale for interpreting the outdoor absolute errors. For the full OURS, the XY APE RMSE corresponds to approximately 0.81%, 2.21%, and 1.49% of the route length on Robot1, Robot2, and Robot3, respectively. The corresponding S-MSCKF ratios are approximately 1.13%, 2.26%, and 2.13%. These ratios show reduced normalized planar error on Robot1 and Robot3, while Robot2 shows a smaller planar change but clearer heading improvement.

**Figure 8:**
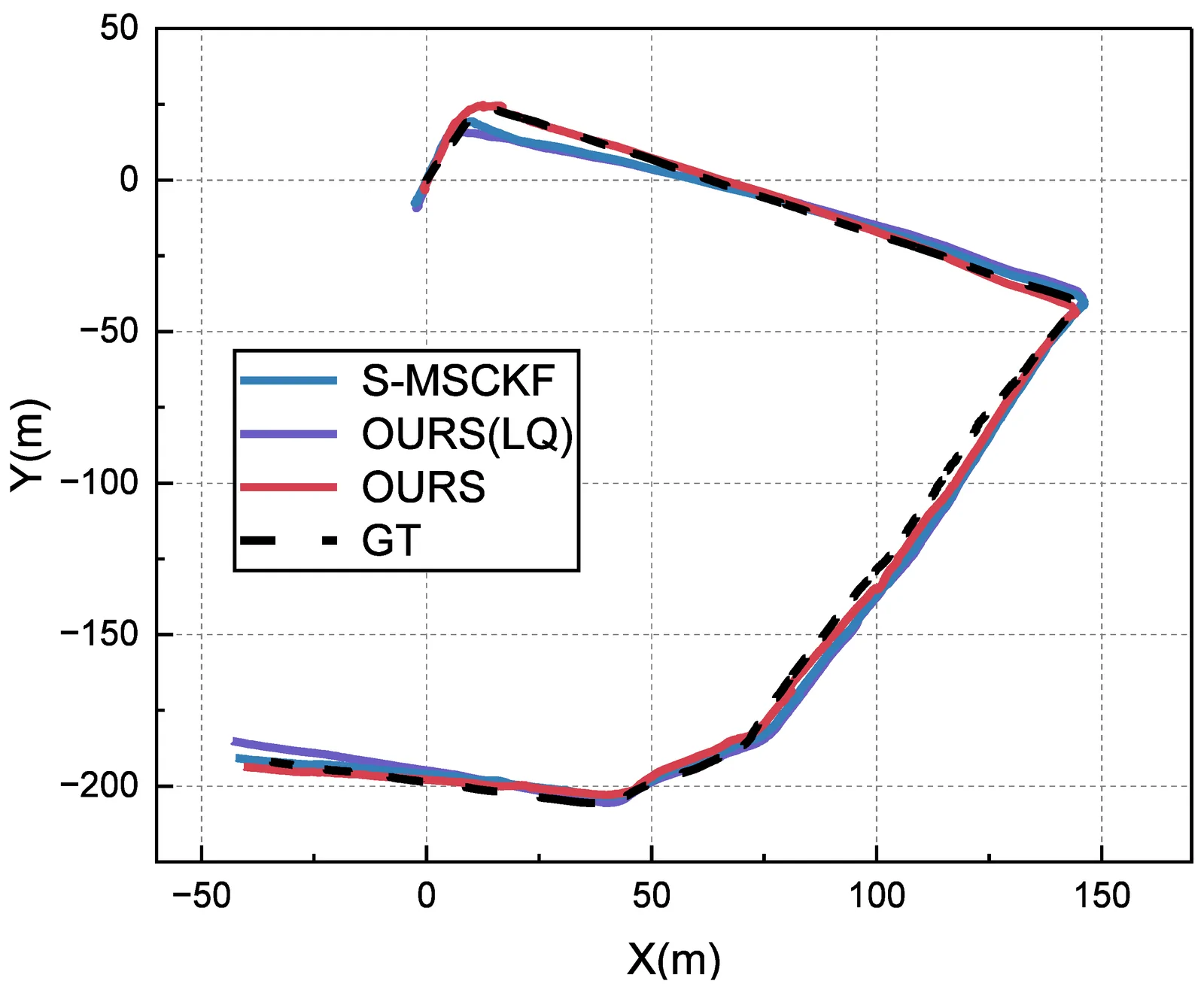
Aligned XY-plane trajectories on the Robot1 sequence.

On *Robot1*, OURS reduces the XY APE RMSE from 5.26 m to 3.76 m, indicating improved planar trajectory consistency. However, the yaw RMSE increases from 3.85° to 4.77°. This behavior is consistent with the geometric role of long parallel structures in outdoor scenes. Road edges, fences, and curb-like boundaries provide strong lateral constraints and can effectively suppress sideward drift in the XY plane. At the same time, line observations are weakly constrained along their own direction; small angular deviations of locally detected parallel segments may contribute to heading bias, an aperture-like ambiguity associated with line features. The aligned XY trajectories in Figure 8 support this interpretation: OURS reduces planar drift relative to the RTK reference, whereas the yaw metric reveals a residual orientation trade-off.

On *Robot2*, which consists of two consecutive square loops, both line-aware variants reduce the yaw RMSE compared with S-MSCKF. OURS(LQ) obtains the lowest XY APE RMSE, whereas the full OURS obtains the lowest yaw RMSE. This indicates a metric-dependent trade-off under outdoor line observations: the full OURS yields slightly larger XY error than OURS(LQ), but obtains the best heading accuracy.

On *Robot3*, the distinction between OURS(LQ) and the full OURS is more pronounced. OURS(LQ) degrades both XY APE RMSE and yaw RMSE relative to S-MSCKF, whereas the full OURS improves both metrics, reducing the XY APE RMSE from 8.13 m to 5.66 m and the yaw RMSE from 8.52° to 7.09°. This contrast provides empirical evidence that the scene-level confidence gate is important for attenuating line-quality-driven intervention when outdoor line cues are unreliable.

Together with the EuRoC results, the three private sequences provide deployment-oriented evidence that the front end is most beneficial when line structures are stable and repeatedly supported by point observations. Under less consistent outdoor line observations, metric-dependent trade-offs between planar position and heading can occur, highlighting the role of scene-adaptive regulation in suppressing adverse line-guidance effects.

### 5.4. Runtime Evaluation

To assess the computational overhead of the line-aware front end, we profiled the front-end runtime on EuRoC MH01, a representative line-rich sequence. The experiment was conducted on an Intel^®^ Core™ i9-13900HX running Ubuntu 20.04. As reported in Table 16, the baseline S-MSCKF front end requires 25.66 ms per frame on average, whereas OURS requires 33.34 ms per frame. This average runtime is close to the 33.3 ms frame period of the 30 Hz stereo input used in the private robot sequences. The μ+3σ runtime of OURS is 47.23 ms, indicating that the upper-tail runtime represented by μ+3σ can exceed a strict 30 Hz frame budget under the tested setting.

**Figure 9:**
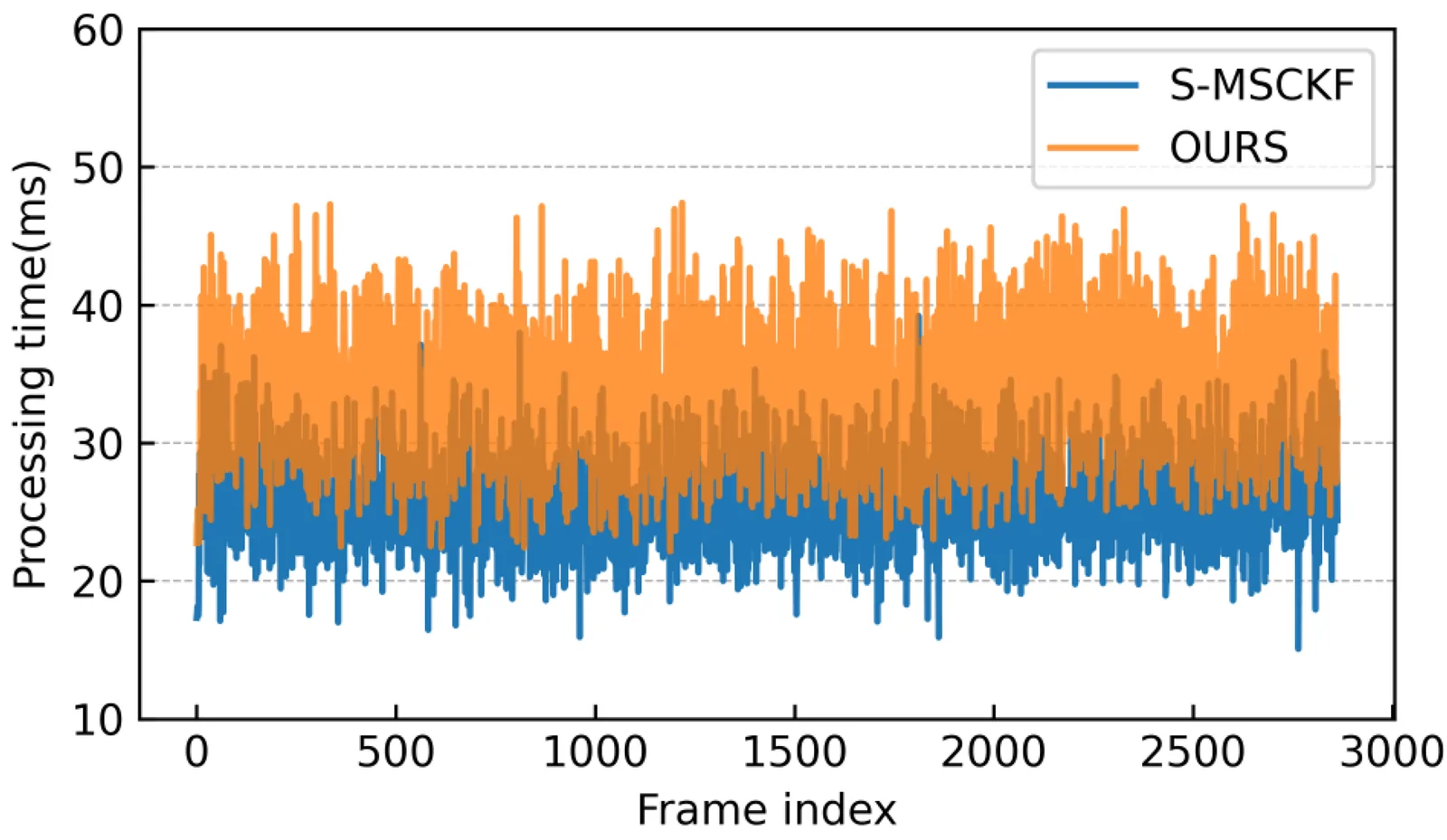
Frame-wise front-end processing time of S-MSCKF and OURS on the EuRoC MH01 sequence.

The line-related time includes line detection, line-quality scoring, point–line association, scene-confidence estimation, and bounded conditioning/pruning. These modules add 8.27 ms per frame on average, accounting for 24.8% of the total front-end runtime. As shown in Figure 9, the additional cost remains moderate compared with the baseline point-tracking and feature-management pipeline. The main overhead comes from image-line extraction and point–line association: the former depends primarily on image resolution and edge content, whereas the latter depends on the number and spatial distribution of tracked points and detected segments. This behavior is consistent with the lightweight front-end design, where the additional computation is confined to image-domain line extraction and local point–line operations.

The same implementation was also used during outdoor data collection with the 30 Hz D435i stereo stream. The runtime results show that the line-aware front end introduces moderate computational overhead and operates near the average timing budget of the tested 30 Hz visual–inertial input, although the μ+3σ runtime leaves limited margin for strict frame-by-frame 30 Hz processing on the tested CPU platform.

## 6. Conclusions

This article presented a scene-adaptive, line-aware visual measurement conditioning method for stereo VIO front ends with point-measurement updates. The method uses 2-D line segments as lightweight structural priors to condition existing point observations before a fixed-interface back-end update. In the evaluated S-MSCKF implementation, the reported EuRoC results show that the sequence-averaged ATE RMSE is reduced by approximately 13% relative to S-MSCKF, with reductions of up to approximately 27% on machine-hall sequences. On the outdoor robot measurement dataset, the mean Sim(2)-aligned planar position error decreases from 8.72 m to 7.31 m, and the mean yaw error decreases from 8.02° to 6.76°. Candidate-level stereo-consistency diagnostics show subpixel mean and 95th-percentile image-domain perturbations without systematic vertical-stereo bias, while the actual primary-view update satisfies the implementation-specific analytical bound in (52).

The experiments also identify boundary conditions of the current design. The V1_02 diagnostics indicate that weak-parallax or visually unstable line support can shorten feature-track persistence even when instantaneous residuals improve. The scale-preserving SE(2) diagnostic shows that the outdoor planar comparison is affected by similarity-scale correction, and the sequence-level results reveal possible position–heading trade-offs under some outdoor line configurations. Runtime profiling indicates moderate computational overhead: the average front-end runtime remains close to the 30 Hz frame budget, but the upper-tail runtime indicates that strict frame-by-frame 30 Hz operation is not guaranteed on the tested CPU platform. The private-data validation is also limited to a single ground-robot platform.

Future work will investigate parallax- or depth-aware line confidence to better distinguish geometrically effective structural support from distant visual line artifacts. Additional directions include conditioning-aware covariance modeling; detector-robust reliability estimation; and evaluation of the same fixed-interface conditioning idea across filtering and optimization-based VIO front ends on aerial, ground, and handheld platforms.

## Figures and Tables

**Table 1:** Comparison with representative related methods.

Method Group	Use of Line/ Structural Cues	Estimator Interface	Scene Adaptivity	Measurement-Integrity Check	Difference from This Work
Point-only VIO front ends [9,10]	No explicit line support; point tracking and grid/lifetime management	Standard point-measurement update	Limited to point-level feature management	Usually focuses on residual gating and tracking quality	Does not exploit persistent point–line support in structured scenes
Explicit point–line VIO/ SLAM [17,18,20,29,30,31]	Line landmarks, line reprojection residuals, or point–line residuals	Requires additional line modeling or residual construction	Mainly estimator- or model-driven	Typically evaluates trajectory accuracy	Improves constraints by changing the estimation model, while this work preserves the point-measurement back-end interface
Measurement-oriented line-feature VIO studies [19,21]	Structural lines for robot localization or adaptive line extraction for point–line VIO	Uses line features within the VIO measurement formulation	Targets line-rich, low-texture, or illumination-changing measurement conditions	Reports localization or measurement-system performance	Motivates line-aware sensing, but does not study bounded point-measurement conditioning under an unchanged point-measurement interface
Point-based front-end adaptation [7,10,32]	Feature quality, tracking history, spatial distribution, or semantic/ static-region cues	Usually preserves point-based updates	Feature- or region-level adaptation	Focuses on outlier suppression or feature quality	Does not explicitly model persistent point–line support or stereo-consistency perturbation
Proposed method	2-D line segments provide image-domain structural support for point observations	Unchanged stereo point-measurement interface in the evaluated S-MSCKF implementation	Segment reliability plus scene-level confidence gate	Provides analytical boundedness, candidate-level stereo-consistency diagnostics, failure diagnostics, and runtime analysis	Converts line support into bounded visual measurement conditioning rather than new line states or residuals

**Table 2:** Principal notation used in the proposed measurement-conditioning formulation.

Notation	Meaning
$z_{k}^{i,\mathrm{raw}}$	Raw stereo point observation for feature *i* in frame *k*
$p_{0,k}^{i,\mathrm{raw}}$	Raw primary-view point coordinate
$p_{1,k}^{i,\mathrm{raw}}$	Raw right-view stereo correspondence
$L_{j,k}$	Detected 2-D line segment
$q_{\mathrm{img},j,k}$	Image-evidence line quality
$q_{\mathrm{line},j,k}$	Support-updated line reliability
$q_{\mathrm{final},j,k}$	Reliability after directional consistency
$w_{\mathrm{scene},k}$	Scene-level line-support confidence
$e_{i,k}$	Signed normal point–line residual
$\phi_{i,k}$	Reliability-dependent intervention strength
$a_{i,k}$	Soft conditioning factor
$\delta_{i,k}$	Final bounded primary-view coordinate displacement
${\tilde{z}}_{k}^{i}$	Conditioned stereo point observation

**Table 3:** Implementation sequence of the proposed line-aware measurement-conditioning layer.

Step	Operation	Output Passed Forward
1	Extract primary-view line segments and image evidence	$L_{k}$, $q_{\mathrm{img},j,k}$
2	Associate raw point observations with segments	$I_{k}^{\mathrm{assoc}}$, $j^{★}\left(i,k\right)$, $d_{i,k}$
3	Update support-driven and directional line reliability	$q_{\mathrm{final},j,k}$
4	Estimate frame-level structural confidence	$w_{\mathrm{scene},k}$
5	Apply bounded conditioning and sparse safeguard	${\tilde{I}}_{k}$, ${\tilde{p}}_{0,k}^{i}$
6	Package standard stereo point measurements	${\tilde{Z}}_{k}$ for the unchanged back-end update

**Table 4:** Sensor configuration of the private robot measurement dataset.

Device	Model	Core Parameters	Freq.
Stereo camera	D435i	848×480; baseline: 50 mm	30 Hz
Built-in IMU	Bosch BMI055 (Bosch Sensortec GmbH, Reutlingen, Germany)	6-axis gyro/accelerometer	200 Hz
RTK-aided INS	CGI-1010	RTK position accuracy: ≤2 cm + 1 ppm (1σ); attitude: 0.01° (1σ); heading: 0.05°; gyro bias: 0.0017rad/h; accel. bias: 30 μg	100 Hz

**Table 5:** Fixed implementation parameters of the proposed front-end layer.

Module	Symbol/Parameter	Value	Role/Selection Basis
Line detection	FLD length/distance/ Canny thresholds; aperture/merging	30 px/1.0 px/80, 200; 3/false	Candidate segment extraction; fixed FLD configuration
Image evidence	$\ell_{\min}$/$s_{g}$/normalization percentile/ Sobel aperture/ length–gradient weights	30 px/10 px/90th/3/0.5, 0.5	Image-level quality normalization
Point–line association	$q_{\mathrm{assoc}}$/$d_{\mathrm{assoc}}$/$d_{\mathrm{good}}$	0.30/4.0 px/1.5 px	Reliable local structural support
Support reliability	α/$\tau_{0}$/support-count scale	0.7/5 frames/90th percentile	Persistent point support weighting
Directional consistency	$B_{\theta }$/$W_{\min}$/$\sigma_{\theta }$/β/$N_{\theta }$	6/0.15/20°/0.8/5	Suppresses unstable orientations
Scene confidence	$\tau_{\min}$/$q_{\min}$/$\lambda_{p}$/η	3 frames/0.60/0.70/0.90	Scene-level support and temporal stabilization
Scene confidence	$s_{\min}$/$s_{\max}$	0.01/0.08	Maps raw support to intervention strength
Bounded conditioning	$\sigma_{\mathrm{px}}$/$\Delta_{0}$/$q_{0}$/γ	1.0 px/0.6 px/0/0.50	Pixel-scale bounded coordinate update
Sparse pruning	$d_{\mathrm{prune}}$/$q_{\mathrm{prune}}$/$w_{\mathrm{prune}}$/ $\tau_{\mathrm{prune}}$/maximum ratio	3.0 px/0.85/0.20/4 frames/3%	Ordered residual gate with 3% maximum removal

*Note:* The fixed implementation parameters explicitly used in the proposed formulation are summarized above. Numerical floors are fixed to $\varepsilon_{\mathrm{norm}}=10^{-3}$ for normalization spans and $\varepsilon_{\deg}=10^{-6}$ for near-zero denominators. Lower-level optical-flow, grid-distribution, stereo-matching, and MSCKF update settings follow the baseline implementation and are kept unchanged.

**Table 6:** ATE RMSE and ARE RMSE on the reported EuRoC sequences. Each entry reports ATE RMSE (m)/ARE RMSE (deg). S-MSCKF and OURS are reported as five-run mean values, while VINS-Mono and ROVIO are external reference systems. V2_03_difficult is excluded following the original S-MSCKF evaluation protocol because the baseline is reported to fail on this sequence owing to stereo feature-matching failure [9].

Method	MH01	MH02	MH03	MH04	MH05	V1_01	V1_02	V1_03	V2_01	V2_02
S-MSCKF	0.15 ^2^/2.22	0.22/1.21	0.25/1.71	0.27 ^2^/1.15 ^2^	0.34/1.34	0.08 ^2^/2.42	0.12 ^2^/1.37 ^2^	0.20/3.55	0.08 ^2^/0.83 ^2^	0.14 ^2^/2.03
VINS-Mono (VIO)	0.19/**1.62**	**0.16**/1.16 ^2^	0.22 ^2^/**0.85**	0.38/1.36	**0.32**/**1.07**	0.08 ^2^/**2.19**	**0.10**/**1.18**	**0.15**/2.63	0.09/2.14	**0.13**/**1.40**
ROVIO	0.37/3.55	0.56/1.91	0.32/2.69	1.12/2.08	1.10/1.92	0.12/2.52	0.18/2.44	0.18/**2.01**	0.30/2.66	0.74/5.54
OURS	**0.11**/1.81 ^2^	0.20 ^2^/**1.07**	**0.19**/1.57 ^2^	**0.21**/**1.14**	0.33 ^2^/1.22 ^2^	**0.07**/2.39 ^2^	0.12 ^2^/1.44	0.16 ^2^/2.55 ^2^	**0.07**/**0.71**	0.14 ^2^/1.70 ^2^
$\Delta_{S\to \mathrm{OURS}}$ ATE	+27%	+9%	+24%	+22%	+3%	+13%	0%	+20%	+13%	0%
$\Delta_{S\to \mathrm{OURS}}$ ARE	+18%	+12%	+8%	+1%	+9%	+1%	−5%	+28%	+14%	+16%

*Note:* Bold values denote the best result for each metric and sequence, and superscript ^2^ denotes the second-best result. The relative changes and ranking markers are determined from the values displayed in the table for consistency with the reported numerical precision. More precise five-run statistics are provided in Table A1. Positive $\Delta_{S\to \mathrm{OURS}}$ values indicate relative error reduction over S-MSCKF. The marker is attached to the corresponding metric value rather than to the whole cell. VINS-Mono and ROVIO are included as external reference systems and are not component-wise baselines of the proposed front-end conditioning layer.

**Table 7:** Run-to-run variability of the controlled EuRoC evaluation.

Method	Mean ATE RMSE (m)	Mean ATE std. (m)	Max. ATE std. (m)	Mean ARE RMSE (deg)	Mean ARE std. (deg)	Max. ARE std. (deg)
S-MSCKF	0.184	0.031	0.079	1.78	0.31	0.64
OURS	0.160	0.029	0.059	1.56	0.26	0.83

*Note:* The standard deviations are computed across five independent runs for each sequence. The table reports the average and maximum of these within-sequence standard deviations over the ten reported EuRoC sequences.

**Table 8:** Component ablation of the proposed front-end layer on the reported EuRoC sequences. ATE RMSE and ARE RMSE are sequence-averaged values over the same ten sequences.

Variant	ATE RMSE	ARE RMSE	$\Delta_{S\to \mathrm{var}}$ ATE	$\Delta_{S\to \mathrm{var}}$ ARE	ATE Increase vs. Full	ARE Increase vs. Full
Full method	**0.160**	**1.56**	+13.0%	+12.4%	–	–
w/o line-quality weighting	0.186	1.76	−1.1%	+1.1%	+16.3%	+12.8%
w/o directional consistency	0.172	1.67	+6.5%	+6.2%	+7.5%	+7.1%
w/o scene confidence	0.162	1.70	+12.0%	+4.5%	+1.3%	+9.0%
Conditioning-focused	0.168	1.65	+8.7%	+7.3%	+5.0%	+5.8%
Pruning-focused	0.177	1.65	+3.8%	+7.3%	+10.6%	+5.8%

*Note:* Bold values indicate the best result in each error metric. Positive $\Delta_{S\to \mathrm{var}}$ values indicate relative error reduction with respect to the sequence-averaged S-MSCKF baseline reported in Table 7. The last two columns report the error increase relative to the full method. The component removed or retained in each variant is defined in the preceding paragraph.

**Table 9:** Sensitivity of the residual conditioning bound $\Delta_{0}$ on the ten reported EuRoC sequences. Mean ATE and mean ARE are sequence-averaged values. Mean standard deviations are computed by first calculating the five-run standard deviation for each sequence and then averaging over the ten sequences.

$\Delta_{0}$	Mean ATE RMSE (m)	Mean ATE std. (m)	Mean ARE RMSE (deg)	Mean ARE std. (deg)
0.4 px	0.168	0.031	1.57	0.32
0.6 px	**0.160**	**0.029**	**1.56**	**0.26**
0.8 px	0.175	0.039	1.65	0.32

*Note:* Bold values indicate the best result in each column.

**Table 12:** Failure-case diagnostics on V1_02.

Metric	OURS	S-MSCKF
Residual RMSE	0.001219	0.001227
Residual P95	0.002308	0.002329
Gating pass ratio	99.04%	98.82%
Mean track lifetime	9.03	10.04
P90 track lifetime	21.64	23.76
Grid occupancy entropy	0.940	0.940
Empty-grid ratio	11.96%	12.09%
Features/grid std.	1.217	1.235

*Note:* Lower residual values indicate cleaner instantaneous point updates, whereas shorter track lifetimes indicate reduced multi-frame persistence.

**Table 13:** Characteristics of the private robot measurement sequences.

Sequence	Route Length	Duration	Approx. 30 Hz Input Frames	Route and Scene Condition
Robot1	466.25 m	358.58 s	10,757	Long straight-line driving with road-edge and fence-like structural support under outdoor illumination variation
Robot2	565.25 m	458.40 s	13,752	Two consecutive square-loop motions with repeated turns and mixed open-area/structured boundaries
Robot3	380.88 m	459.75 s	13,793	Outdoor route with sparser or less persistent line support, vegetation-side edges, and illumination changes

*Note:* The approximate input-frame counts are derived from the recorded duration and the 30 Hz D435i stereo input. The RTK-aided INS reference accuracy is listed in Table 4.

**Table 14:** Planar position and heading errors on the private robot dataset (XY APE RMSE/yaw RMSE, m/deg). Route length is given in metres. Bold values indicate the best result for each metric on each sequence.

Seq.	Length	S-MSCKF	OURS(LQ)	OURS
Robot1	466.25	5.26/**3.85**	5.69/4.54	**3.76**/4.77
Robot2	565.25	12.76/11.70	**12.14**/8.56	12.52/**8.43**
Robot3	380.88	8.13/8.52	9.16/10.85	**5.66**/**7.09**
Mean	–	8.72/8.02	9.00/7.98	**7.31**/**6.76**

**Table 15:** Scale-preserving outdoor diagnostic under SE(2) alignment. Each method cell reports SE(2)-aligned XY APE RMSE/yaw RMSE (m/deg). The listed $s^{★}$ is the scale factor estimated by the corresponding Sim(2) alignment in Table 14; values away from one indicate that the Sim(2) comparison includes noticeable scale correction. Bold values indicate the best result for each metric on each sequence.

Seq.	S-MSCKF	OURS(LQ)	OURS
Robot1	14.52/3.85 ($s^{★}$ = 1.163)	**14.18**/4.54 ($s^{★}$ = 1.155)	16.06/4.77 ($s^{★}$ = 1.193)
Robot2	13.39/11.70 ($s^{★}$ = 1.114)	**12.79**/8.56 ($s^{★}$ = 1.111)	13.15/**8.43** ($s^{★}$ = 1.111)
Robot3	**8.54**/8.52 ($s^{★}$ = 1.055)	10.54/10.85 ($s^{★}$ = 1.106)	9.16/**7.09** ($s^{★}$ = 1.157)

**Table 16:** Front-end runtime breakdown on the EuRoC MH01 sequence.

Config.	Avg. (ms)	Std. (ms)	μ+3σ (ms)	Line-Time (ms)	Line-Time Ratio
S-MSCKF	25.66	3.32	35.62	–	–
OURS	33.34	4.63	47.23	8.27	24.8%

## Data Availability

The EuRoC MAV dataset used in this study is publicly available. The private robot-sequence data that support the findings of this study are available from the corresponding authors upon reasonable request.

## Appendix A

**Table A1:** Per-sequence run-to-run variability over five independent EuRoC runs.

Seq.	S-MSCKF ATE	OURS ATE	S-MSCKF ARE	OURS ARE
MH_01	0.145±0.024	0.109±0.027	2.22±0.32	1.81±0.15
MH_02	0.216±0.040	0.195±0.059	1.21±0.18	1.07±0.18
MH_03	0.251±0.027	0.191±0.042	1.71±0.16	1.57±0.22
MH_04	0.269±0.061	0.206±0.033	1.15±0.33	1.14±0.26
MH_05	0.343±0.079	0.331±0.044	1.34±0.60	1.22±0.28
V1_01	0.080±0.018	0.071±0.015	2.42±0.11	2.39±0.10
V1_02	0.116±0.004	0.121±0.018	1.37±0.39	1.44±0.33
V1_03	0.201±0.027	0.164±0.028	3.55±0.64	2.55±0.83
V2_01	0.076±0.010	0.071±0.011	0.83±0.20	0.71±0.10
V2_02	0.144±0.017	0.142±0.015	2.03±0.18	1.70±0.14

*Note:* ATE values are in meters and ARE values are in degrees. The means reproduce the five-run S-MSCKF and OURS entries reported in Table 6 before rounding.
